# Gene therapy for polygenic or complex diseases

**DOI:** 10.1186/s40364-024-00618-5

**Published:** 2024-09-04

**Authors:** Tingting Wu, Yu Hu, Liang V. Tang

**Affiliations:** 1 Institute of Hematology, Union Hospital, Tongji Medical College, Huazhong University of Science and Technology, Wuhan, 430022 China; 2 Key Laboratory of Biological Targeted Therapies of the Chinese Ministry of Education, Wuhan, China

**Keywords:** Gene therapy, Polygenic diseases, Complex diseases, CRISPR-CAS9

## Abstract

Gene therapy utilizes nucleic acid drugs to treat diseases, encompassing gene supplementation, gene replacement, gene silencing, and gene editing. It represents a distinct therapeutic approach from traditional medications and introduces novel strategies for genetic disorders. Over the past two decades, significant advancements have been made in the field of gene therapy, leading to the approval of various gene therapy drugs. Gene therapy was initially employed for treating genetic diseases and cancers, particularly monogenic conditions classified as orphan diseases due to their low prevalence rates; however, polygenic or complex diseases exhibit higher incidence rates within populations. Extensive research on the etiology of polygenic diseases has unveiled new therapeutic targets that offer fresh opportunities for their treatment. Building upon the progress achieved in gene therapy for monogenic diseases and cancers, extending its application to polygenic or complex diseases would enable targeting a broader range of patient populations. This review aims to discuss the strategies of gene therapy, methods of gene editing (mainly CRISPR-CAS9), and carriers utilized in gene therapy, and highlight the applications of gene therapy in polygenic or complex diseases focused on applications that have either entered clinical stages or are currently undergoing clinical trials.

**Graphical Abstract:**

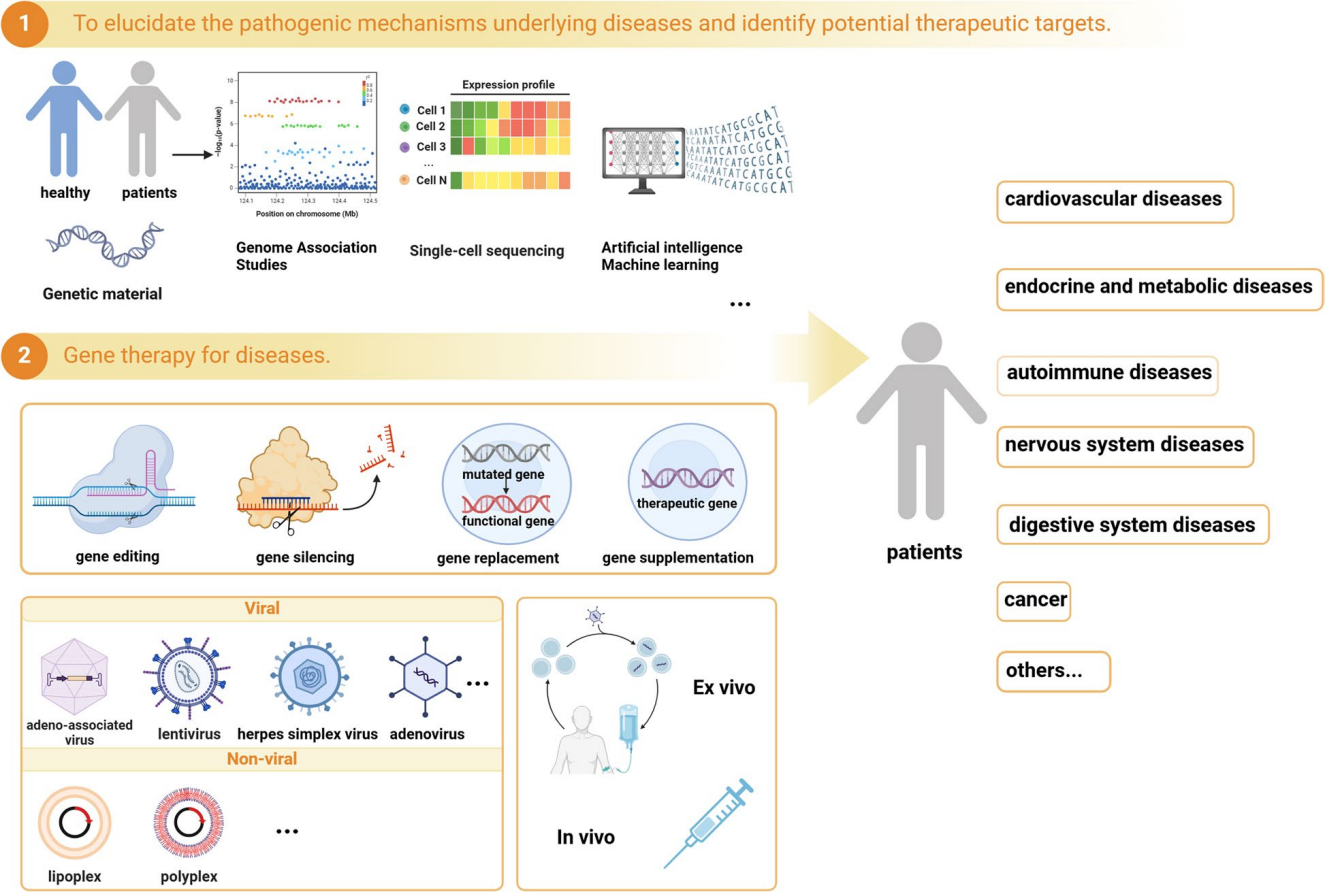

## Introduction

Gene therapy employs nucleic acid-based therapeutics to address human diseases encompassing gene replacement for defective genes in patients, silencing deleterious mutated genes within cells (using siRNA, miRNA or antisense oligonucleotides), overexpressing genes via gene supplementation, and rectifying the patient's genome through gene editing, thereby achieving the goal of disease treatment [1–5]. Gene therapy has emerged as a promising alternative for diseases unresponsive to conventional pharmaceutical interventions, showcasing remarkable potential in the treatment of various ailments, particularly those with hereditary origins. In contrast to traditional drugs, gene therapy represents a "causative therapeutic approach," aiming not only to provide transient symptom relief but also to achieve sustained expression of therapeutic genes, ultimately striving for long-term prevention, treatment, and even eradication of diseases. The first successful gene therapy, which is considered a milestone achievement, employs ex vivo gene therapy to treat severe combined immunodeficiency (SCID) caused by adenosine deaminase (ADA) deficiency. This therapeutic approach involves the infusion of autologous T cells that have been genetically modified using a recombinant retrovirus carrying the ADA gene, aiming to restore the patient's immune function [6]. The initial focus of gene therapy was primarily directed towards monogenic diseases and cancer. Significant breakthroughs have been accomplished in the field of monogenic diseases, leading to successive approvals and market availability of gene therapy products. These involve a wide range of disorders affecting multiple systems, including hematological conditions (hemophilia A and B [7, 8], sickle cell anemia, β-thalassemia [9]), neurological disorders (spinal muscular atrophy [10], duchenne muscular dystrophy [11], aromatic L-amino acid decarboxylase deficiency [12]), and vision loss [13]. Moreover, with the advancement of gene sequencing technology, more associations between diseases and genes have been revealed. This enhanced understanding and research into the molecular pathological mechanisms underlying diseases have established a foundation for personalized gene therapy. The success of gene therapy has also facilitated its expansion into the field of non-monogenic or complex diseases.

This review aims to discuss the strategies of gene therapy, methods of gene editing, and carriers utilized in gene therapy. Additionally, it will explore the application of gene therapy in non-monogenic or complex disorders such as cardiovascular diseases, neurodegenerative diseases, autoimmune diseases, and endocrine/metabolic disorders. The primary focus will be on applications that have advanced to clinical trials or are presently implemented within clinical settings.

### Basic gene therapy strategies

Gene therapy involves two fundamental strategies [14]. Firstly, by integrating the drug gene into stem cells or precursor cells, it can be transmitted to the daughter cells after division. Secondly, through the utilization of non-integrating vectors, the drug gene can be transferred to quiescent or slowly dividing cells, thereby achieving sustained expression of the drug-gene throughout the cellular lifespan. The non-integrating approach becomes more applicable when the expression of transgenes in post-mitotic cells can serve therapeutic purposes.

### Approaches to gene editing

The advancement of gene therapy is intricately linked to the support provided by gene editing technology. Currently, the commonly employed gene editing techniques encompass clustered regularly interspaced short palindromic repeats associated nuclease 9(CRISPR-CAS9), transcription activator-like effector nucleases (TALENs), and zinc finger nucleases (ZFNs). CRISPR-CAS9 was initially discovered within the bacterial natural immune system and exploits the CRISPR-Cas system to precisely cleave the target DNA sequence by pairing Cas9 with specific gRNA. This enables genome modification through the cell's inherent repair mechanism. Notably, CRISPR-CAS9 represents a straightforward, highly efficient, and user-friendly gene editing technique [15, 16]. TALENs constitutes an artificial protein complex comprising transcriptional activation factors and nucleases that facilitate DNA cleavage for genome manipulation via cellular repair mechanisms [17]. ZFNs consist of zinc finger proteins responsible for precise recognition of DNA sequences coupled with nucleases that execute targeted DNA cleavage [18]. Additionally, there exist several innovative approaches to gene editing. The prime editing technique enables precise modifications of DNA sequences, including base substitutions, insertions, and deletions, without requiring double-stranded DNA breaks (DSBs) or donor DNA. This system comprises a Cas9 nickase fused with an engineered reverse transcriptase and utilizes the prime editing guide RNA (pegRNA) to achieve targeted gene editing [19]. Base editing is a CRISPR-based technology that facilitates precise modifications to specific base pairs on DNA or RNA without inducing DSBs [20]. The Bridge RNAs direct programmable genome-editing system is based on a bispecific non-coding RNA expressed by the IS110 family of mobile genetic elements that enables the precise insertion, excision, or inversion of specific target DNA sequences [21].

### Ex vivo and in vivo gene therapy

#### Ex vivo

For ex vivo applications, the process typically involves three steps: isolation of target cells from the patient's body, in vitro genetic engineering of these cells, and subsequent autologous transplantation to reintroduce the modified cells back into the patient's body. The modified target cells will continue to express gene of interest, thus achieving the goal of treatment. Due to the minimal patient harm and the knowledge gained from bone marrow transplantation, blood cells have emerged as the primary target cells, including hematopoietic stem cells and mature blood cells [22, 23]. The utilization of T lymphocytes as target cells has become predominant in the study of mature blood cells. In 1990, a clinical trial employed retroviral transduction of the ADA gene into T cells as a therapeutic approach for children with ADA-SCID, restoring their immune responses. Over two years, gene therapy demonstrated sustained expression of the ADA gene [24]. Over the past decade, chimeric antigen receptor (CAR)-T cell therapy has emerged as a highly promising immunotherapeutic approach for combating cancer. Engineered immune cells express antigen receptors capable of recognizing and eliminating tumor cells. Genetically modified immune cells are redirected towards tumor cells via chimeric antigen receptors (CARs), which reprogram the patient's T cells to effectively eradicate malignant neoplastic growth [25]. Although CAR is categorized as a form of cell therapy, it exhibits overlapping characteristics with gene therapy.

**Fig. 1:**
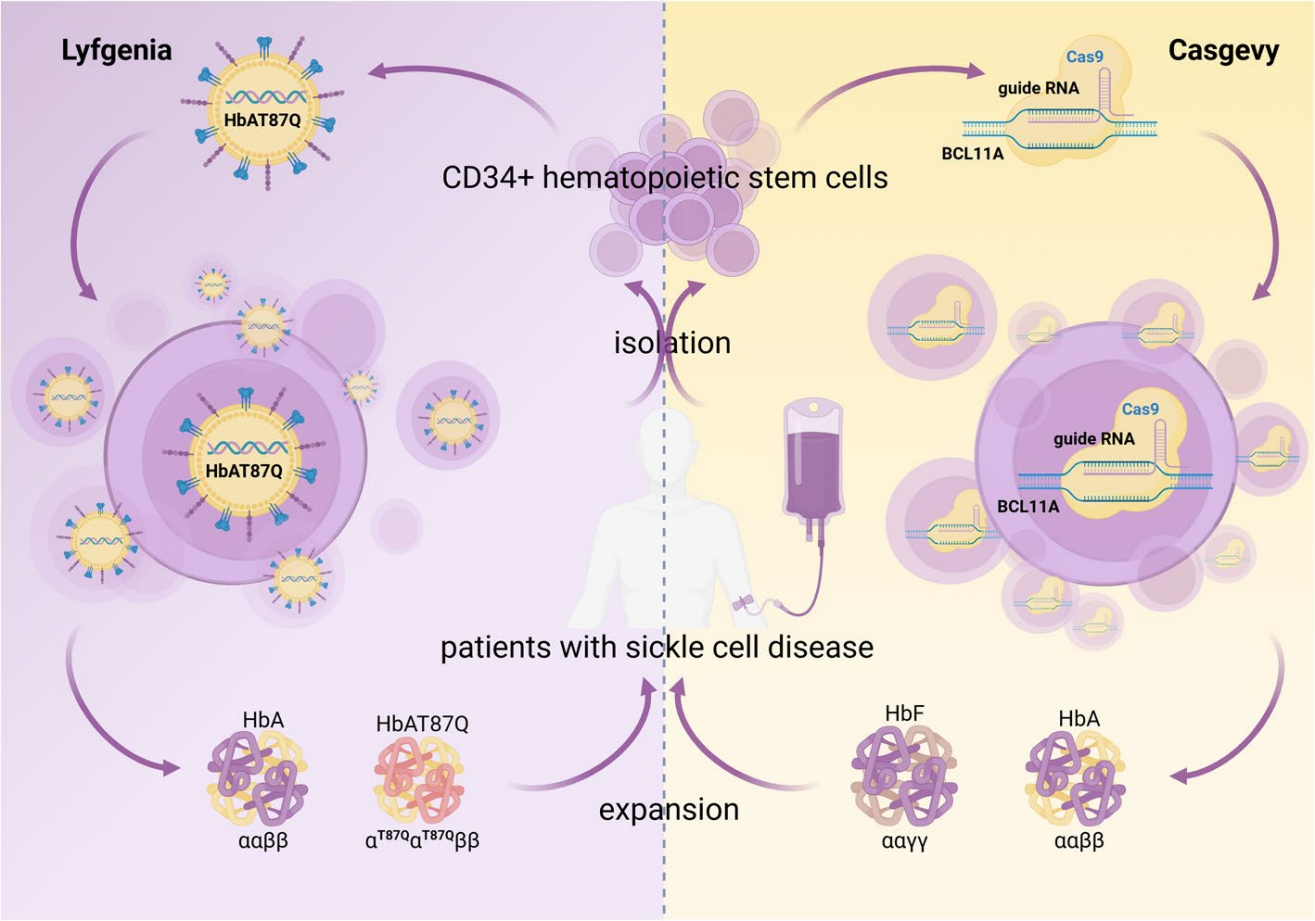
To illustrate the ex vivo gene therapy approach, we employ sickle cell anemia as a paradigm for gene therapy. Casgevy: Initially, CD34 + hematopoietic stem and progenitor cells were isolated from the patient. Precise gene editing was performed on the patient's hematopoietic stem cells by specifically targeting the erythroid enhancer region of BCL11A using a single-guide RNA molecule (sgRNA). Subsequently, the edited cells were reintroduced into the patient's body with the aim of combating sickle cell anemia through increased expression of HbF. Lyfgenia: The BB305 lentiviral vector was employed to transduce modified β-globin genes into hematopoietic stem cells, resulting in the production of HbAT87Q—a hemoglobin variant that is resistant to disease and capable of inhibiting sickle hemoglobin polymerization. The remaining steps remain consistent with those described by Casgevy. Created with BioRender.com

The American Food and Drug Administration (FDA) approved two gene therapies, Casgevy (exagamglogene autotemcel) and Lyfgenia (lovotibeglogene autoemcel), for the treatment of sickle cell disease (SCD) on December 8th, 2023 [26, 27]. This milestone represents the inaugural authorization of cell-based gene therapy for patients aged 12 and above with sickle cell disease in the United States. Notably, Casgevy is the first therapeutic intervention utilizing CRISPR-Cas9 gene editing technology to receive FDA approval (Fig. 1). SCD is caused by mutations in the β-globin chain gene of hemoglobin, affecting a global population exceeding three million individuals [28]. BCL11A is a transcription factor responsible for the repression of fetal hemoglobin (HbF) expression. After mobilization of the bone marrow, CD34 + hematopoietic stem cells and progenitor cells were harvested from the patient. The erythroid enhancer region of BCL11A was precisely targeted using a single-guide RNA molecule (sgRNA) for precise gene editing on the patient's hematopoietic stem cells. After undergoing a series of myeloablative treatments, the edited target cells were reinfused, resulting in increasing production of HbF [9]. According to the currently available clinical trial data (NCT03745287 and NCT04208529), the proportion of HbF in total hemoglobin (Hb) after administration of Casgevy treatment was 43.9% at the sixth month, and this level was sustained throughout the observation period (at least 24 months). The gene therapy approach employed by Lyfgenia, which also falls within the ex vivo pathway, differs in its treatment strategy (Fig. 1). It utilizes the BB305 lentiviral vector to transduce modified β-globin genes into hematopoietic stem cells, resulting in the production of HbAT87Q, a disease-resistant hemoglobin that can counteract sickle hemoglobin polymerization [29]. As of February 13, 2023, data from the Phase 1/2 HGB-206 Group C (NCT02140554) and Phase 3 HGB-210 studies (NCT04293185) revealed that complete resolution of severe vaso-occlusive events (sVOE) and VOE was observed in a significant proportion of evaluable patients, with rates reaching 94% (32/34) and 88% (30/34), respectively, during the five-year follow-up period (median duration: 35.5 months; range: 0.3–61 months), encompassing a total of 47 patients.

In addition to the readily obtainable blood cells, the combination of induced pluripotent stem cells (iPSCs) and gene editing technology offers an alternative approach for ex vivo gene therapy. The advent of iPSCs and their derivatives, which emerged 16 years ago, has provided a valuable source of human cells for diverse applications encompassing drug discovery, toxicity assessment, disease modeling, and personalized cell therapy [30]. The synergistic utilization of iPSCs in conjunction with gene editing technology has established a solid groundwork for tailored cellular therapeutics [31]. By isolating peripheral blood mononuclear cells (PBMCs) from patients and subjecting them to gene reprogramming, it is possible to induce their conversion into pluripotent stem cells. Subsequently, these pluripotent stem cells can be genetically modified to rectify the patient's defective genes. Through controlled differentiation conditions, the pluripotent stem cells can be directed toward specific cell lineages and subsequently reintroduced into the patient's body, thereby accomplishing therapeutic objectives [32].

#### In vivo

The in vivo approach, as opposed to the ex vivo gene therapy delivery method, bears a greater resemblance to the conventional administration of pharmaceutical agents. These include peripheral intravenous infusion (systemic administration) or local infusion (such as portal vein, renal vein, intrathecal injection), targeted tissue administration (local injection into specific brain regions, subretinal injections), and peripheral intramuscular injection [33–35]. The research objective of in vivo gene therapy is to enhance the precision, efficacy, and long-term effectiveness of gene therapy. Presently, the majority of approved gene therapy drugs predominantly employ in vivo administration.

### Vectors for the delivery of gene therapy

The critical step in gene therapy entails the delivery of therapeutic genes to target cells and tissues. The carriers utilized for gene delivery include viral vector platforms as well as non-viral vector platforms (Table 1).

**Table 1:** Advantages and disadvantages of viral and non-viral vectors

**Viral platforms**			
	**Advantages**	**Disadvantages**	**Approved drugs**
			**Drugs/targets/diseases**
Adenovirus	• Large cargo capacities (~ 36 kb) • High transduction efficiency in both quiescent and dividing cells • Non-integrative within the host genome	• Transient expression • Widespread neutralizing antibodies • Stronger immunological response	• Gendicine/ p53 gene/ head and neck cancer • Adstiladrin/ Interferon alfa-2b / NMIBC • Oncorine/ oncolytic / e.g. head and neck cancer; hepatocarcinoma; cervical cancer
AAV	• Minimal immunogenicity • Longer transgene expression • Transduce multiple tissue/cell types	• Small cargo capacities (~ 4.7 kb)	• Luxturna/ RPE65 / IRD • Glybera/ LPL / LPLD • Zolgensma/ SMN1 / SMA • Upstaza/ AADC/AADC deficiency • Roctavian/ B domain deleted hFVIII/HA • Elevidys/ micro-dystrophin protein / DMD • Hemgenix/ FIX-Padua /HB
Lentivirus	• Long-term transgene expression • Medium cargo capacities (~ 8 kb) • Powerful vector for in vitro genetic modification	• Integrative within the host genome	• Strimvelis/ autologous CD34 + HSPCs /ADA-SCID • Zynteglo/ autologous CD34 + HSPCs /β-Thalassemia • Libmeldy/ autologous CD34 + HSPCs / MLD • Skysona/ autologous CD34 + HSPCs/ CALD • Kymriah/ autologous T cells/ ALL;DLBCL
HSV	• Large cargo capacities (~ 30 kb) • Safer • Neurotropic	• Low titers • Complex production process	• Lmlygic/ GM-CSF / melanoma • Delytact/ oncolytic/ glioblastoma • Vyjuvek/ COL7A1 / DEB
**Non-viral platforms**			
	**Advantages**	**Disadvantages**	**Investigational drugs**
			**Vectors/ targets/diseases**
e.g Nanoparticles Cationic- polymers Liposomes	• Wide raw materials • Non-pathogenic agents • Easy to modify • High nucleic acid loading • Safety • Lower toxicity and cost	• Lower delivery efficacies • Transient expression	• PEI/ DTA-H19/ Bladder cancer(NCT00595088) • Lipid nanoparticle/ VEGF and KSP/ advanced solid tumors with liver involvement(NCT00882180) • PEG Nanocomplex CALAA-01/ M2 subunit of ribonucleotide reductase / Solid tumor cancers(NCT00689065) • Cationic lipids DOTAP-cholesterol/ FUS1/ Non-small cell lung cancer(NCT00059605)

*NMIBC* Non-muscle-invasive bladder cancer, *AAV* Adeno-associated virus, *RPE65* Retinal pigment epithelium 65 kDa protein, *IRD* Inherited retinal degeneration, *LPL* Lipoprotein lipase, *LPLD* Lipoprotein lipase deficiency, *SMN1* Survival motor neuron 1, *SMA* Spinal muscular atrophy, *AADC* Aromatic amino acid decarboxylase, *hFVIII* Human coagulation factor VIII, *HA* Hemophilia A, *DMD* Duchenne muscular dystrophy, *FIX* Coagulation factor IX, *HB* Hemophilia B, *HSPCs* Hematopoietic stem and progenitor cells, *ADA-SCID* Adenosine deaminase-severe combined immunodeficiency, *MLD* Metachromatic leukodystrophy, *CALD* Cerebral adrenoleukodystrophy, *ALL* Acute lymphoblastic leukemia, *DLBCL* Diffuse large B-cell lymphomas, *HSV* Herpes simplex virus, *GM-CSF* Granulocyte–macrophage colony-stimulating factor, *COL7A1* The gene encoding the anchoring fibril component, collagen VII (C7), *DEB* Dystrophic epidermolysis bullosa, *PEI* Polyethylenimine, *VEGF* Vascular endothelial growth factor, *KSP* Kinesin spindle protein, *PEG* Polyethylene glycol, *DOTAP* N-[1-(2,3-Dioleoyloxy) propyl]-N,N,N-trimethylammonium methyl-sulfate, *FUS1* Tumor suppressor

### Viral vectors

The structural components of viral vectors for gene therapy consist of three essential elements. Firstly, the protein capsid or envelope determines the specific recognition and affinity towards target tissue cells. Secondly, the gene of interest, when expressed within the cell, achieves the desired therapeutic effect. Lastly, the regulatory cassette comprising enhancers, promoters, and other regulatory elements is responsible for precisely regulating either stable or transient expression of the gene of interest. Presently, the most widely accepted and extensively employed viral vector platforms primarily revolve around adenoviruses, lentiviruses, adeno-associated viruses (AAV), and herpes simplex virus (HSV).

Adenoviruses is a non-enveloped, linear, double-stranded DNA virus. The adenoviruses utilized in contemporary gene therapy are derived from human adenovirus serotypes 2 and 5. Through genetic engineering and modification of the wild-type adenovirus genome, specific structures are substituted with transgenes, resulting in a series of adenovirus-based gene therapy vectors. Adenovirus, as a gene therapy vector, possesses significant advantages owing to its robust packaging capacity reaching 36 Kb in a number of modifications. Moreover, adenovirus-based vectors exhibit high transduction efficiency in both quiescent and dividing cells, displaying a wide tropism for different tissue types. Importantly, they remain non-integrative within the host genome. However, the widely pre-existing viral immunity in the population and potent immunogenicity of adenovirus impose limitations on its applicability in gene therapy. Presently, it is predominantly employed in innovative vaccine development and cancer treatment [36, 37].

AAV is a single-stranded DNA virus that harbors four known open reading frames. It is currently acknowledged as non-pathogenic and has not been linked to any diseases [38]. Its packaging capacity is approximately 4.7 kb. Due to its limited packaging capacity, a dual-vector system has been developed to facilitate efficient genome packaging [39]. AAV demonstrates a diverse range of tissue and cell tropism, attributed to the existing AAV serotypes and modifications in the viral capsid. Additionally, the incorporation of tissue-specific promoters has significantly enhanced its tropism [40]. In comparison to other viral vectors, AAV is acknowledged for its minimal immunogenicity. Numerous clinical trials based on this platform have proliferated rapidly.

Lentivirus is classified as a single-stranded RNA retrovirus, characterized by its long-term integrated vector and packaging capacity of approximately 9 kb. It serves as an efficient platform for gene-modified cell therapy, allowing the expression of multiple genes using a single vector. In vitro gene modification heavily relies on the utilization of lentivirus [41].

Naturally, the HSV primarily spreads through direct contact, predominantly in the perioral region. It typically manifests as a relatively benign ailment; however, in rare instances, it can invade the central nervous system and cornea, precipitating grave consequences such as encephalitis and visual impairment. Engineered HSV-1 has emerged as a promising vehicle for gene therapy due to its neurotropic properties, rendering it potentially valuable in addressing neurological disorders [42]. Furthermore, its robust packaging capacity confers an advantageous attribute. In May 2023, Vyjuvek received approval in the United States. It employs the HSV-1 vector to deliver the COL7A1 gene encoding collagen type VII, which is employed for treating dystrophic epidermolysis bullosa. Moreover, it represents a breakthrough as the first FDA-approved gene therapy product for repeated administration, administered topically in gel form [43].

### Non-viral vector

Currently, approximately 70% of clinical trials employ viral vectors. However, the utilization of viral vectors is accompanied by certain limitations such as transgenic insertional mutations, immunogenicity, and intricate preparation procedures, which impose restrictions on their applications [44–46]. Consequently, the exploration of non-viral vectors has exhibited remarkable potential and necessity. Compared to viral vectors, non-viral vectors exhibit superior nucleic acid loading capacity and safety profiles, as well as offering greater flexibility in terms of chemical composition and a broader range of raw material options. Non-viral vectors encompass a diverse range of delivery systems, including lipid nanoparticles (LNPs), exosomes, cationic polymers such as (PAE), single-chain cyclic polymer (SCKPs), polyethyleneimine (PEI), poly(amidoamine) (PAMAM), polydimethylaminoethyl methacrylate (PDMAEMA), chitosan (CS), and cyclodextrin (CD), inorganic nanoparticles, and intelligent hydrogels. Notably, LNPs and cationic polymers have gained significant traction in various applications [47, 48]. Recently, Zhang Feng's team has discovered a programmable protein delivery method that utilizes bacterial contractile injection systems (eCISs). These eCISs are natural protein delivery systems similar to an "injector" found in bacteria. The tail fibers of the eCISs have been modified to enable them to target specific cells and carry various proteins such as Cas9 and base editor proteins. Furthermore, modifications to other components of this system could potentially allow for the delivery of RNA or DNA [49]. This system shows promise as a safe and efficient tool for gene therapy in the future.

### Gene therapy for non-monogenic disorders/complex diseases

Over the past two decades, there has been a growing ease in identifying the causal genes for highly penetrant, Mendelian (monogenic) human diseases. These diseases, characterized by their low occurrence rate, are commonly classified as orphan diseases. In contrast to monogenic diseases, polygenic diseases are associated with multiple gene mutations or single nucleotide polymorphisms. The impact of these variants is relatively smaller and the underlying mechanism for complex diseases lies in the accumulation of subtle effects on key genes and regulatory pathways that contribute to disease risk. The advent of Genome-Wide Association Studies (GWAS) has provided optimism in identifying single polymorphic variants with discernible functional impacts on complex traits. Polygenic risk scores serve as indicators of an individual's susceptibility to disease [50, 51]. Polygenic diseases encompass a group of prevalent age-related conditions such as cardiovascular disease, diabetes, and cancer. Additionally, early or middle-aged onset complex polygenic disorders like asthma, Crohn's disease, schizophrenia, systemic lupus erythematosus have emerged as current areas of research interest [52]. Differing from monogenic disorders, management strategies for polygenic diseases not only focus on symptom treatment but also emphasize meaningful early preventive measures to mitigate further damage caused by disease progression. Selective clinical trials for gene therapy in non-monogenetic diseases are summarized in Table 2.

**Table 2:** Selective clinical trials for gene therapy in non-monogenetic diseases

Trial	Vector	Target gene	Approach	Design	n	Follow-up time	Primary endpoint	Main result	Reference
**Cardiovascular system**									
**CAD**									
VIF-CAD	plasmid	VEGF-A165/bFGF	NOGA-guided i. my. injections	phase 2	52	median, 133 months	safety	safe	[53]
Symes JF et al.	plasmid	VEGF165	i. my. injections	phase 1	20	180 days	safety; bioactivity	safe; positive	[54]
Kołsut P et al.	plasmid	VEGF165	i. my. injections	-	22	-	safety; efficacy	safe; positive	[55]
Tio RA et al.	plasmid	VEGF165	NOGA-guided i. my. injections	-	35	-	appraise the value of PET in the assessment of the effect	positive	[56]
Rosengart TK et al.	adenovirus	VEGF121	i. my. injections	phase 1	31	median, 11.8 years	safety	safe	[57]
Rosengart TK et al.	adenovirus	VEGF121	i. my. injections	phase 1	21	-	safety; efficacy	safe; positive	[58]
Lathi KG et al.	plasmid	VEGF165	i. my. injections	phase 1	30	180 days	clinical changes in cardiovascular function	negative	[59]
the NOVA trial	adenovirus	VEGF121	NOGA-guided i. my. injections	-	17	52 weeks	safety; efficacy	safe; negative	[60]
Hassinen I et al.	adenovirus	VEGF-D^∆N∆C^	combined NOGA and PET mapping; NOGA-guided i. my. injections	phase 1/2a	30	12 months	efficacy of combining electromechanical map with PET imaging to target the ischemic myocardium	positive	[61]
Sarkar N et al.	plasmid	VEGF-A165	i. my. injections	Open-labelled study	39	12 months	safety; bioactivity	safe; may be positive	[62]
Stewart DJ et al.	adenovirus	VEGF121	i. my. injections	phase 2	67	26 weeks	efficacy	positive	[63]
Yang ZJ et al.	adenovirus	HGF	over the wire balloon or by diagnostic coronary catheter	phase 1	18	14 months	safety	safe	[64]
Meng H et al.	Adenovirus	HGF	percutaneous i. my. injections	phase 2a	30	6 months	safety; efficacy	safe; potentially efficient in improving LVEF and lowering LVDd	[65]
Losordo DW et al.	plasmid	VEGF	i. my. injections	phase 1	5	-	safety; bioactivity	safe; positive	[66]
Vale PR et al.	plasmid	VEGF165	i. my. injections	-	13	-	efficacy	positive	[67]
KAT	Adenovirus, plasmid	VEGF	catheter-based local intracoronary gene transfer	phase 2	103	6 months	safety; feasibility	no differences in clinical restenosis rate or minimal lumen diameter; a significant increase was detected in myocardial perfusion in the VEGF-Adv–treated patients	[68]
Euroinject One	plasmid	VEGF165	NOGA-guided i. my. injections	phase 2	80	3 months	efficacy	not significantly improve stress-induced myocardial perfusion abnormalities; improved regional wall motion	[69]
NORTHERN	plasmid	VEGF165	NOGA-guided i. my. injections	-	93	6 months	efficacy	negative	[70]
Ripa RS et al.	plasmid	VEGF-A165	i. my. injections	-	16	3 months	safety; effects	negative	[71]
AGENT	adenovirus	FGF-4	percutaneous i. my. injections	-	79	mean,311 days	safety; effects	safe; positive	[72]
Grines CL et al.	adenovirus	FGF-4	intracoronary injections	-	52	12 months	safety; effects	safe; positive	[73]
**PAD**									
Kim HJ et al.	plasmid	VEGF165	i. m. injections	phase 1	9	9 months	safety; clinical effects	safe; positive	[74]
Niebuhr A et al.	plasmid	FGF1	i. m. injections	phase 1 and 2	93	3 years	safety	safe	[75]
pUDK-HGF	plasmid	HGF	i. m. injections	phase 2	240	-	safety; efficacy	safe; positive	[76]
Barć P et al.	plasmid	VEGF165/HGF	i. m. injections	-	28	90 days	efficacy	positive	[77]
Rajagopalan S et al.	adenovirus	VEGF	i. m. injections	phase 2	105	26 weeks	efficacy	negative	[78]
Makino H et al.	plasmid	HGF	i. m. injections	phase 1/2a	22	2 years	efficacy	positive	[79]
Kusumanto YH et al.	plasmid	VEGF165	i. m. injections	-	54	100 days	efficacy	negative	[80]
STOP-PAD	plasmid	SDF-1	i. m. injections	phase 2b	109	3 months	safety; efficacy	safe; negative	[81]
Deev R et al.	plasmid	VEGF165	i. m. injections	-	36	5 years	safety; efficacy	safe; positive	[82]
Rajagopalan S et al.	adenovirus	VEGF121.10	i. m. injections	phase 1	6	30 days	safety; efficacy	safe; positive	[83]
Mohler ER 3rd et al.	adenovirus	VEGF121.10	i. m. injections	phase 1	15	360 days	safety	safe	[84]
NL003	plasmid	HGF	i. m. injections	phase 2	200	6 months	safety; efficacy	safe; positive	[85]
DVC1-0101	rSeV	FGF-2	i. m. injections	phase 1/2a	12	6 months	safety; efficacy	safe; positive	[86]
Cui S et al.	plasmid	HGF	i. m. injections	phase 1	21	3 months	safety; preliminary efficacy	safe; positive	[87]
VM202	plasmid	HGF	i. m. injections	phase 1	12	12 months	safety	safe	[88]
pCK-HGF-X7	plasmid	HGF	i. m. injections	phase 1	21	3 months	safety; preliminary efficacy	safe; positive	[89]
CI-1023	adenovirus	VEGF121.10	i. m. injections	phase 1	18	1 year	safety; efficacy	safe; encouraging trends in ABI at rest and peak walking time	[90]
**HF**									
SERCA-LVAD trial	AAV1	SERCA2a	intracoronary infusion	phase 2a	5	3 years	safety; feasibility	safe; negative	[91]
STOP-HF	plasmid	SDF-1	endomyocardial injections	phase 2	93	12 months	safety; efficacy	safe; negative	[92]
CUPID	AAV1	SERCA2a	intracoronary infusion	phase 2	39	12 months	safety; efficacy	safe; positive	[93]
CUPID 1	AAV1	SERCA2a	intracoronary infusion	phase 1/ 2	39	3 years	clinical effects	positive	[94]
CUPID 2	AAV1	SERCA2a	intracoronary infusion	phase 2b	250	median 17.5 months	efficacy	negative	[95]
Hammond HK et al.	adenovirus 5	AC6	intracoronary infusion	phase 2	56	1 year	safety; efficacy	safe; positive	[96]
AGENT-HF	AAV1	SERCA2a	intracoronary infusion	phase 2	9	6 months	safety; efficacy	safe; negative	[97]
**Hyperlipidemia**									
ORION-10 ORION-11	siRNA-based drug	PCSK9	s. c. injections	phase 3	1561;1617	540 days	reductions in LDL cholesterol levels (in patients with atherosclerotic cardiovascular disease and or an atherosclerotic cardiovascular disease risk equivalent)	approximately 50% reductions	[98]
ORION-9	siRNA-based drug	PCSK9	s. c. injections	phase 3	482	540 days	reductions in LDL cholesterol levels (in patients with heterozygous familial hypercholesterolemia)	approximately 39% reductions	[99]
ORION-1	siRNA-based drug	PCSK9	s. c. injections	phase 2	501	240 days	reductions in LDL cholesterol levels	dose-dependent reductions	[100]
ORION-3	siRNA-based drug	PCSK9	s. c. injection	-	497	4 years	results from the 4-year open-label extension of the ORION-1 trial	averaged mean reduction of LDL-C cholesterol was 44.2%	[101]
ORION-5	siRNA-based drug	PCSK9	s. c. injection	phase 3	56	150 days	reductions in LDL cholesterol levels (in patients with homozygous familial hypercholesterolemia)	negative	[102]
OCEAN[a]-DOSE	siRNA-based drug	Lipoprotein(a)	s. c. injection	-	281	36 weeks	the percent change in the lipoprotein(a) concentration from baseline to week 36	dose-dependent manner; -70.5% with the 10-mg dose, -97.4% with the 75-mg dose, -101.1% with the 225-mg dose administered every 12 weeks, and -100.5% with the 225-mg dose	[103]
Yeang C et al.	oligonucleotide drug	apo(a)	s. c. injection	phase 2b	286	12 months	the percent change in the Lp(a)-C concentration	compared with placebo, pelacarsen resulted in dose-dependent decreases in Lp(a)-C (2% vs -29% to -67%)	[104]
Gaudet D et al.	ASO	ANGPTL3	s. c. injection	phase 2	105	6 months	percent change in fasting triglycerides from baseline at 6 months	reductions in triglycerides of 36%, 53%, 47%, were observed in the 40mgQ4W, 80mgQ4W, and 20mgQW groups, respectively	[105]
TRANSLATE-TIMI 70	ASO	ANGPTL3	s. c. injection	phase 2b	286	36 weeks	placebo-adjusted percentage change from baseline in non-HDL-C at 24 weeks	ranging from 22.0% in the 60 mg every 2 weeks arm to 27.7% in the 80 mg every 2 weeks arm	[106]
The APPROACH trial	ASO	APOC3	s. c. injection	phase 3	66	52 weeks	percentage change in fasting triglyceride levels from baseline to 3 months	77% decrease in mean triglyceride levels	[107]
The COMPASS trial	ASO	APOC3	s. c. injection	phase 3	114	-	percentage change from baseline to 3 months in fasting triglyceride	reduced mean plasma triglyceride concentration by 71.2%	[108]
**Hypertension**									
The KARDIA-1	siRNA-based drug	hepatic angiotensinogen	s. c. injection	phase 2	394	6 months	between-group difference in least-squares mean (LSM) change from baseline to month 3 in 24-h mean ambulatory SBP	-14.1 mm Hg (150 mg, once every 6 months); -16.7 mm Hg (300 mg, once every 3 months or every 6 months); and -15.7 mm Hg (600 mg, once every 6 months)	[109]
AGMG0201	vaccine	angiotensin II	i. m. injections	phase 1/2a	24	360 days	safety	tolerated	[110]
**Neurodegenerative diseases**									
**Alzheimer's disease**									
Rafii MS et al.	AAV2	NGF	stereotactically guided intracerebral injections into the nucleus basalis of Meynert	phase 2	49	24 months	change from baseline on the Alzheimer's Disease Assessment Scale-cognitive subscale at month 24	negative	[111]
CERE-110	AAV	NGF	stereotactically guided intracerebral injections into the nucleus basalis of Meynert	open-label	10	2 years	safety and tolerability, and initial efficacy	feasible, tolerated, and able to produce long-term, biologically active NGF expression	[112]
LGT	AAV	hTERT	Ongoing, NCT04133454						
UCSD-BDNF1	AAV2	BDNF	Ongoing, NCT05040217						
LX1001-01	AAVrh.10	APOE2	Ongoing, NCT03634007						
**Parkinson's disease**									
Kaplitt MG et al.	AAV	GAD	unilateral, subthalamic injections	phase 1	12	12 months	safety, tolerability, and potential efficacy	safe; significant improvements in motor UPDRS scores	[113]
CERE-120	AAV2	NTN	bilateral, stereotactic, intraputaminal injections	phase 1	12	1 year	safety, tolerability, and potential efficacy	partially positive	[114]
Marks WJ Jr et al.	AAV2	NTN	bilateral, putaminal injections	phase 2	58	12 months	change of the UPDRS motor score	negative	[115]
Muramatsu S et al.	AAV	AADC	putaminal injections	phase 1	6	6 months	safety, tolerability, and potential efficacy	safe; positive	[116]
Warren Olanow C et al.	AAV2	NTN	bilateral, putaminal and nigral injections	-	51	24 months	change of the UPDRS motor score	negative	[117]
Niethammer M et al.	AAV2	GAD	bilateral, subthalamic injections	-	45	12 months	long-term clinical outcome	positive	[118]
Mittermeyer G et al.	AAV2	AADC	bilateral, putaminal injections	phase 1	10	4 years	long-term clinical outcome	positive	[119]
**Amyotrophic lateral sclerosis**									
ALSpire	ASO-based drug	ATXN2	ongoing, NCT04494256						
**Endocrine and metabolic systems**									
**Diabetes**									
GPX-002	AAV	Pdx1/MafA	remains at the preclinical stage						
VCTX210	stem cell therapy with CRISPR-Cas9 gene editing	B2M, PD-L1, HLA-E	undergoing phase 1 clinical trials and administration to the first patient has been completed						
**Others**									
**Osteoarthritis**									
GNSC-001	AAV	IL-1Ra	intra-articular injections	ongoing, NCT05835895					
**Systemic lupus erythematosus**									
Andreas Mackensen et al.	CAR-T	CD19	autologous T cells reinfusion	-	5	-	clinical efficacy	highly effective	[120]
	CAR-NK	CD19	ongoing, NCT06010472						
	CAR-T	CD19 and BCMA	ongoing, NCT05858684						
	CAR-T	CD19 and BCMA	ongoing, NCT05474885						
	CAR-T	CD19	ongoing, NCT06056921						

*CAD* Coronary artery disease, *i. my*. Intramyocardial, *PET* Positron emission tomography, *LVEF* Left ventricular ejection fraction, *LVDd* Left ventricular end-diastolic dimension, *PAD* Peripheral arterial disease, *rSeV* Recombinant Sendai virus, *ABI* Ankle brachial index, *HF* Heart failure, *s. c*. Subcutaneous, *LDL* Low density lipoprotein, *ASO* Antisense oligonucleotide, *SBP* Systolic blood pressure, *UPDRS* Unified Parkinson's Disease Rating Scale

### Cardiovascular diseases

#### Coronary heart disease

**Fig. 2:**
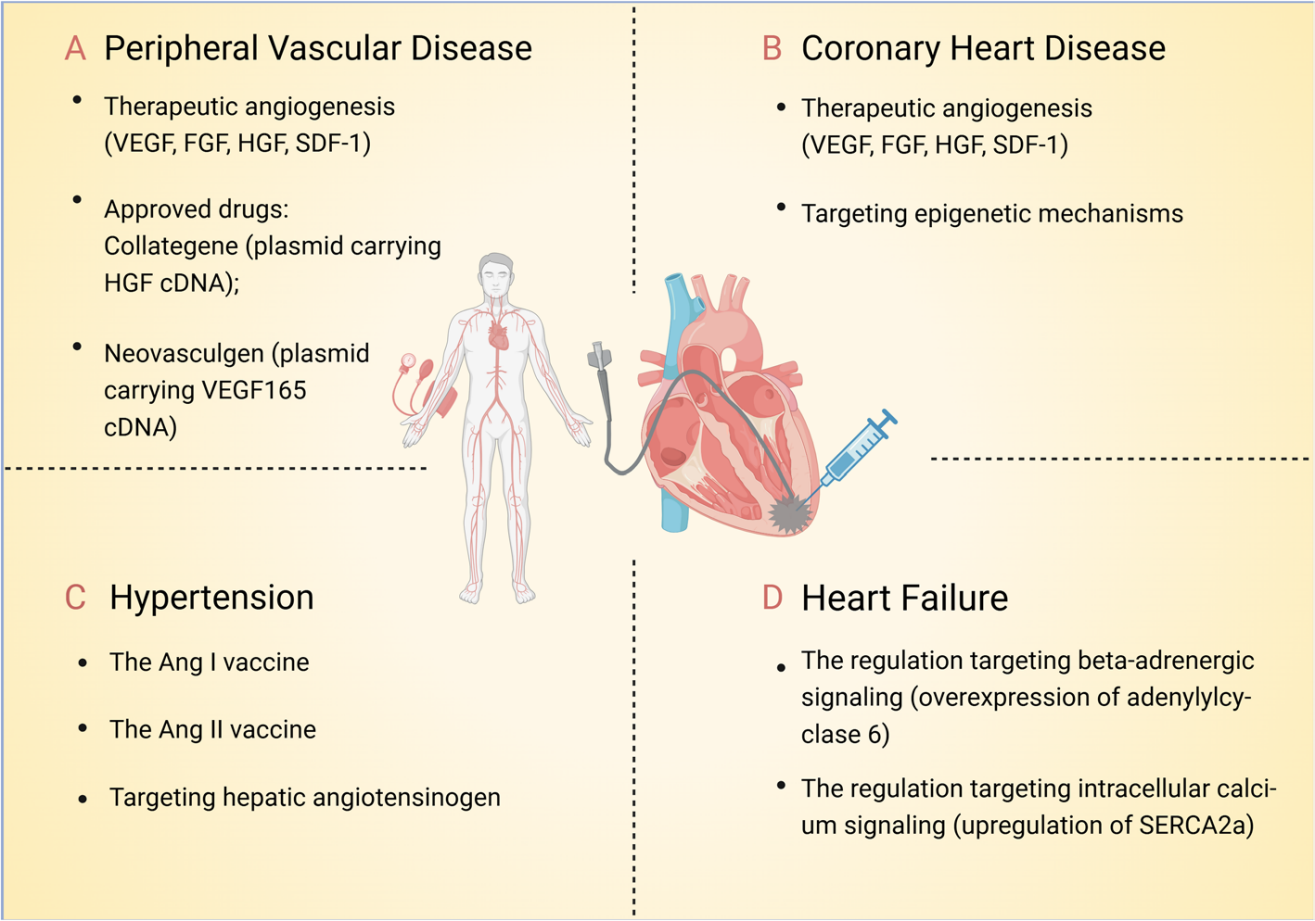
Gene therapy strategies for cardiovascular diseases. VEGF: Vascular endothelial growth factor; FGF: Fibroblast growth factor family; HGF: Hepatocyte growth factor; SDF-1: Stromal cell-derived factor 1; Ang: Angiotensin; SERCA2a: Sarcoplasmic/endoplasmic reticulum Ca2 + ATPase. Created with BioRender.com

Coronary artery disease (CAD) is primarily attributed to the progressive narrowing of the coronary arteries caused by atherosclerosis, consequently leading to ischemic heart disease. Over the past two decades, there have been several endeavors to apply gene therapy in the treatment of CAD. The therapeutic approaches employed in clinical trials for CAD primarily revolve around promoting therapeutic angiogenesis (Fig. 2). This involves utilizing a vector to deliver the cDNA of specific cell factors that stimulate angiogenesis, targeting the affected myocardial region through direct intramyocardial or intra-arterial injection. Commonly utilized factors include vascular endothelial growth factor (VEGF), fibroblast growth factor family (FGF), and hepatocyte growth factor (HGF), stromal cell-derived factor 1 (SDF-1) with VEGF being extensively investigated. Compared to the direct utilization of protein preparations, the gene therapy-mediated protein generation pathway holds the potential to enhance therapeutic protein levels in a more sustained and stable manner. The initial clinical trial for CAD was conducted in 1998 by Douglas W. Losordo et al. [121]. In this study, the researchers administered a direct injection of a naked plasmid (phVEGF165) encoding VEGF into the ischemic myocardium. Following treatment, a significant reduction in angina incidence was observed among patients, which was further confirmed by single-photon emission computed tomography demonstrating decreased myocardial ischemia. Subsequently, a series of clinical trials were conducted employing Ad or naked plasmid vectors (PI), including KAT301 (Ad, VEGF-D^∆N∆C^), ASPIRE (Ad, FGF-4), Haoyu Meng et al. (Ad, HGF), AFFIRM (Ad, FGF-4) (NCT02928094), the VEGF-Neupogen trial (PI, VEGF-A165-hGCSF), VIF-CAD (PI, VEGF-A165/bFGF), and NORTHERN (PI, VEGF-A165) [53, 65, 70, 122–124]. Based on completed or ongoing clinical trials, although some have yielded positive results, demonstrating the safety and effectiveness of gene therapy based on therapeutic angiogenesis in the treatment of CAD, overall, the treatment outcomes remain unsatisfactory. The limited transduction efficiency of the vector in myocardial tissue and the transient duration of transgene expression may be key factors impeding therapeutic efficacy. Furthermore, methodological considerations such as stronger placebo effects in experimental design and a lack of sensitive efficacy evaluation indicators also pose challenges to the advancement of gene therapy for CAD [125]. Currently, there are no drugs that have received market approval.

Recently, studies have demonstrated the pivotal role of epigenetics in cardiovascular diseases. Epigenetic mechanisms, including DNA methylation, histone modifications, and non-coding RNA, intricately regulate genes associated with disease pathology. Furthermore, therapeutic interventions targeting epigenetic regulatory pathways have been developed to address cardiovascular disorders. Although these drugs are currently in the preclinical or clinical trial stage, this treatment approach offers an additional potential avenue for disease management [126].

#### Peripheral vascular disease

Peripheral arterial disease (PAD) is caused by the occlusion of major arteries in the lower extremities as a result of atherosclerosis, leading to limb ischemia and a range of clinical manifestations including pain, claudication, and ulcers. Similar to the treatment of CAD, therapeutic angiogenesis emerges as a key treatment strategy for PAD. FGF has been investigated in this context through trials such as Comerota et al. [127], TALISMAN [128], and TAMARIS [129], utilizing plasmids as vectors. Morishita et al. [130], HGF-STAT [131], and Powell et al. [132] employed plasmid vectors as well; however, their distinction lies in the specific targeting of HGF. RAVE [133], Deev, et al. [134], and other experiments used adenovirus as a vector carrying VEGF cDNA. It is noteworthy that two treatment drugs have received approval for the management of PAD. Collategene [135], a gene therapy product carrying the HGF gene on a plasmid, was granted approval by the Japan Ministry of Health, Labour and Welfare in 2019. This therapeutic approach targets occlusive arteriosclerosis and thromboangiitis obliterans cases that do not respond to standard drug therapy. Furthermore, Neovasculgen [136], based on plasmid-VEGF165, obtained regulatory clearance in Russia back in 2011.

#### Heart failure

With advancements in the understanding of heart failure pathogenesis, several crucial targets implicated in disease onset and progression have been identified. These targets exhibit a characteristic resistance to conventional pharmacological interventions; however, gene therapy has emerged as a promising approach. Various transgenic strategies primarily focus on three key areas: angiogenesis, intracellular calcium signaling regulation, and β-adrenergic signaling. Sarcoplasmic/endoplasmic reticulum Ca2 + ATPase (SERCA2a) serves as the primary calcium pump in cardiomyocytes, facilitating the recycling of calcium ions within the sarcoplasmic reticulum. Its pivotal role in maintaining a stable intracellular calcium ion concentration is indispensable. The upregulation of SERCA2a has shown promising potential in enhancing cardiac function, rendering it an attractive target gene for heart failure therapy [137]. The CUPID 2 trial enrolled a cohort of 250 patients diagnosed with heart failure, who underwent a single coronary artery infusion of AAV1-SERCA2. Regrettably, the administration of AAV1-SERCA2 in this clinical trial did not yield any discernible improvement in the overall clinical trajectory of patients suffering from heart failure [138]. Stromal cell-derived factor-1(SDF-1) is a chemotactic protein that binds to the CXCR-4 receptor, thereby promoting angiogenesis and facilitating tissue regeneration. The STOP-HF trial administered a naked DNA plasmid encoding human SDF-1 (pSDF-1) to patients with heart failure via endocardial injection. Although the efficacy of pSDF-1 was not established by the STOP-HF trial, its data substantiated the safety profile of this therapeutic approach [92]. Adenylyl cyclase (AC) catalyzes the conversion of adenosine triphosphate into cyclic adenosine monophosphate (cAMP). AC6, an isoform found in cardiac muscle cells, plays a crucial role. Dysfunction of the β-adrenergic signaling system is a significant pathological mechanism contributing to heart failure. Targeting overexpression of AC to elevate cAMP levels may present a promising therapeutic approach for treating heart failure. This Phase 2 clinical trial aims to investigate the efficacy of Ad5 encoding adenylyl cyclase 6 (Ad5.hAC6). At week 4, the treatment group exhibited a significant improvement in ejection fraction (EF) (+ 6.0 [1.7] EF units; *n* = 21; *P* < 0.004); however, by week 6, the results demonstrated a lack of sustained effect [139]. These unsatisfactory findings suggest that gene therapy for heart failure has encountered an impasse.

#### Hypertension

DNA or RNA-based vaccines initially emerged in the field of infectious diseases. In 2023, scientists Katalin Karikó and Drew Weissman were honored with the Nobel Prize in Physiology or Medicine for their groundbreaking discoveries in nucleotide modification, which paved the way for the development of highly effective mRNA vaccines against COVID-19. The modification of nucleotide bases can prevent the body from recognizing synthesized mRNA as foreign, thereby significantly expediting the clinical application of mRNA vaccines. During the COVID-19 pandemic, BNT162b2 (Comirnaty) and mRNA-1273 (Spikevax) are two representative mRNA-based vaccines [140]. DNA vaccines are constructed using recombinant plasmids that encode viral antigens. Upon transduction into host cells, these plasmids facilitate the production of proteins or peptides through transcription and translation processes. The concept of developing a vaccine against hypertension involves substituting the encoding sequence for the viral antigen with that of the self-antigen. Firstly, angiotensin I (Ang I) emerges as the primary target for self-directed intervention. In this randomized double-blind placebo-controlled clinical trial, the Ang I vaccine PMD3117 was administered to hypertensive patients; however, the findings did not demonstrate a significant reduction in blood pressure [141]. Given the potential involvement of the renin and Ang II feedback pathway in its failure, researchers have redirected their attention towards Ang II. The Ang II vaccine has demonstrated promising results in preclinical studies [142]. The AGMG0201 trial, a phase I/IIa trial, aims to assess the safety, tolerability, and immunological response of the modified angiotensin II DNA vaccine (AGMG0201). Notably, patients in the AGMG0201 group exhibited detectable levels of antibodies against angiotensin II, particularly among those receiving higher doses [110]. However, further clinical efficacy needs to be substantiated through additional rigorous clinical trials. Zilebesiran, an RNA interference therapeutic targeting hepatic angiotensinogen synthesis, demonstrated significant reduction in 24-h mean ambulatory systolic blood pressure (SBP) at month 3 in adults with mild to moderate hypertension during the phase 2 study, when administered at various doses and intervals of either 3 or 6 months (NCT04936035).

#### Dyslipidemia

**Fig. 3:**
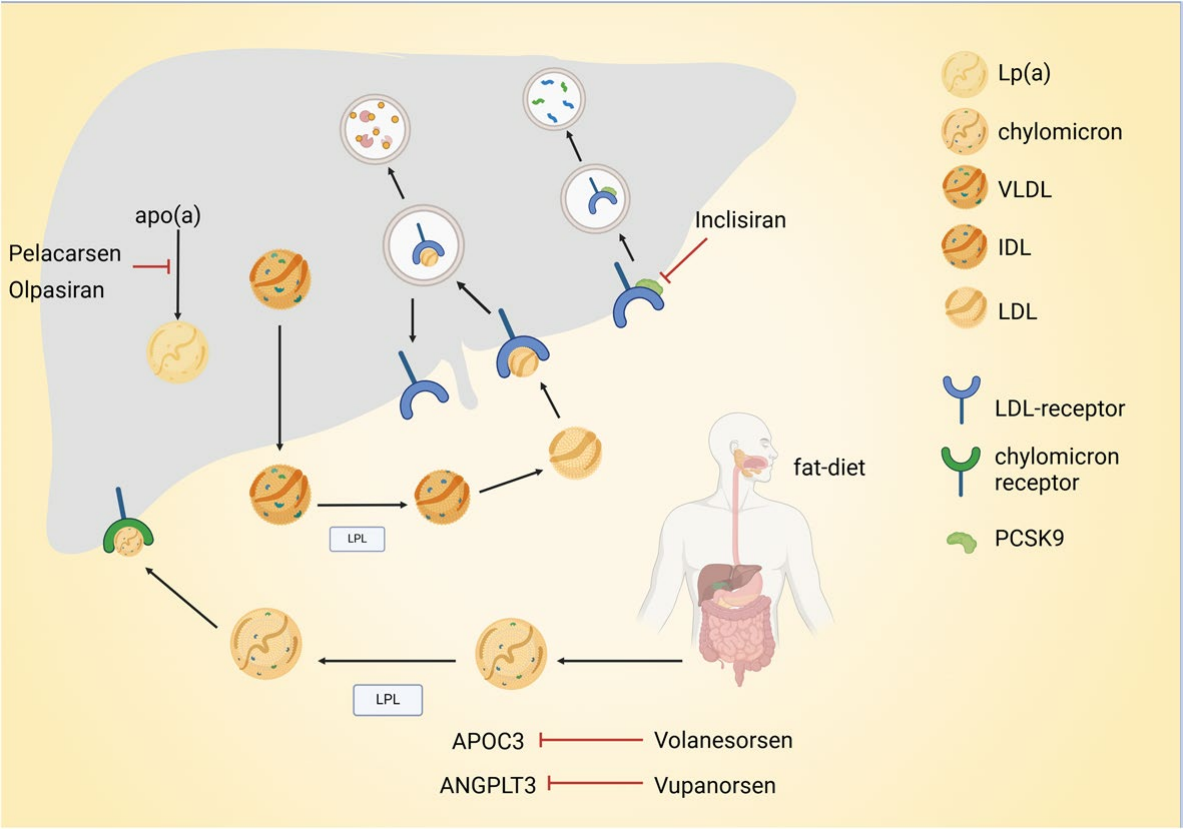
The targets for gene therapy in the treatment of hyperlipidemia. PCSK9, acting as a partner of the LDL receptor, facilitates the trafficking of the LDL receptor to the lysosome, thereby enhancing its degradation. Inclisiran is a siRNA-based drug targeting PCSK9, it hampers the degradation process of the LDL receptor and promotes its recycling instead, ultimately leading to reduced levels of LDL. Olpasiran is a siRNA-based agent that effectively impedes the hepatic assembly of Lp(a) by downregulating its expression in hepatocytes, Pelacarsen is an oligonucleotide drug that inhibits the synthesis of apo(a), resulting in a dose-dependent reduction in Lp(a) levels. Vupanorsen and Volanesorsen are ASO drugs targeting ANGPTL3 and ApoC-III respectively. Lp(a): Lipoprotein(a); VLDL: Very low-density lipoprotein; IDL: Intermediate-density lipoprotein; LDL: Low-density lipoprotein; PCSK9: Proprotein convertase subtilisin/kexin type 9; APOC3: Apolipoprotein C-III; ANGPTL3: Angiopoietin-like protein; ASO: Antisense oligonucleotides; LPL: Lipoprotein lipase. Created with BioRender.com

Hyperlipidemia is a well-established risk factor for CAD, with low-density lipoprotein (LDL) being closely related to the development of coronary atherosclerosis. Consequently, reducing LDL levels has emerged as the primary objective of lipid-lowering therapy. In recent years, advancements in genetic analyses have unveiled novel targets, including proprotein convertase subtilisin/kexin Type 9 (PCSK9), angiopoietin-like protein (ANGPTL3), apolipoprotein C-III (ApoC-III), and Lipoprotein(a) (Lp(a)) (Fig. 3). The objective of lipid-lowering therapies is to achieve optimal efficacy and long-term durability. The development of lipid-lowering treatment drugs is progressing from traditional oral medications (administered daily) to monoclonal antibody drugs (given monthly or semi-monthly), oligonucleotide drugs (administered weekly or monthly), siRNA drugs (administered semi-annually), and beyond, with extended durations being explored [143]. Antisense oligonucleotides (ASOs) are chemically modified fragments of single-stranded DNA or RNA molecules, utilized in the treatment of human diseases through mechanisms such as translational inhibition, mRNA degradation, and regulation of splicing. The RNA interference pathway, mediated by small double-stranded RNA molecules (dsRNA), promotes mRNA degradation and downregulates gene expression, inducing gene-silencing effects [144]. PCSK9, acting as a partner of the LDL receptor, facilitates the trafficking of the LDL receptor to the lysosome, thereby enhancing its degradation. In cases where PCSK9 function is impaired, it hampers the degradation process of the LDL receptor and promotes its recycling instead, ultimately leading to reduced levels of LDL. Inclisiran is a siRNA-based drug targeting PCSK9. The ORION-3 study is a four-year open-label extension conducted in five countries, demonstrating an average reduction of 44.2% (95% CI: 47.1 ~ 41.4) and 62% ~ 77.8% in LDL-C levels and PCSK9 levels respectively over the four-year observation period. The incidence of possibly treatment-related serious adverse reactions was found to be 1%. These findings provide robust evidence supporting the sustained efficacy and favorable tolerability profile of biannual Inclisiran injections for LDL-C lowering over a prolonged duration [101]. Previously, the ORION-9 [99], ORION-10 [98], and ORION-11 [145] trials elucidated the lipid-lowering efficacy and tolerability of Inclisiran over a duration of up to 18 months. Olpasiran is also a siRNA-based agent that effectively impedes the hepatic assembly of Lp(a) by downregulating its expression in hepatocytes, thereby resulting in reduced levels of Lp(a). The phase 2 OCEAN[a]-DOSE trial demonstrates that a subcutaneous injection of Olpasiran at a dosage of 75 mg or higher every 12 weeks leads to a remarkable reduction in Lp(a) levels exceeding 95% after a follow-up period of 36 weeks. Notably, all patients (100%) receiving either the 75 mg or the 225 mg dose of Olpasiran every 12 weeks achieved normal Lp(a) levels [103]. Pelacarsen (AKCEA-APO(a)-LRx, IONIS- APO(a)-LRx, TQJ230) is an oligonucleotide drug that inhibits the synthesis of apo(a), resulting in a dose-dependent reduction in Lp(a) levels up to 80% [146]. Currently, phase III clinical trials are ongoing (NCT04023552). Additionally, Vupanorsen and Volanesorsen have demonstrated promising results as ASO drugs targeting ANGPTL3 and ApoC-III respectively [105, 107, 108]. Furthermore, in vivo, gene editing of PCSK9 in primates has shown potential for long-term lipid reduction by lowering LDL for at least 8 months [147].

### Central nervous system diseases

#### Alzheimer’s disease

Alzheimer's disease (AD) is a multifaceted neurodegenerative disorder that affects more than 50 million individuals globally, predominantly the elderly population [148]. AD is characterized by the abnormal accumulation of amyloid-beta (Aβ) in the cerebral cortex and hippocampus as well as the aberrant formation of neurofibrillary tangles, leading to progressive memory loss and cognitive impairment. Currently, the underlying pathological mechanisms of AD remain incompletely elucidated. Hypotheses regarding its pathogenesis encompass elevated levels of tau proteins, diminished antioxidant capacity, impaired cholinergic activity, and neural inflammation, among others. Moreover, through advancements in GWAS and epidemiological investigations, numerous genes potentially implicated in AD have been identified, including APOE, TREM2, FERMT2, APP, etc.; these genes are involved in a diverse array of proteins directly or indirectly associated with AD [148]. Human apolipoprotein E (APOE) is considered the most significant genetic factor associated with late-onset AD, with APOE having three alleles ε2, ε3, and ε4. Among these alleles, ε4 encodes apoE4, which confers a lifetime risk estimate of developing AD by the age of 85 at approximately 30% in apoE4 heterozygotes and approximately 65% in apoE4 homozygotes. However, APOE encoded by ε2 is believed to exert a protective effect. Under physiological conditions, APOE primarily facilitates lipid and cholesterol transport and metabolism while also playing a crucial role in neuronal maintenance and repair. ApoE4 promotes Aβ production while impeding its degradation, induces abnormal hyperphosphorylation of tau protein, and affects neuroinflammatory cell function and activation, ultimately contributing to the pathogenesis of AD [149]. This ongoing phase 1/2 open-label study (NCT03634007) aims to employ intrathecal administration of serotype AAVrh.10 gene transfer vector expressing the cDNA encoding human apolipoprotein E2 (APOE2) to evaluate the therapeutic efficacy of this intervention in individuals with AD who are homozygous for APOE4. Preclinical safety assessments have been conducted using non-human primates [150]. The Nerve Growth Factor (NGF), an endogenous neurotrophic factor with neuroprotective properties, represents another target for AD gene therapy. AAV2-NGF was administered via stereotactic intracerebral injection into the basal ganglia in a study (NCT00876863). However, regrettably, no significant difference was observed between the treatment and placebo groups based on the Alzheimer Disease Assessment Scale–cognitive subscale [151]. Given the association between late-onset AD and aging, this ongoing study (NCT04133454) aims to employ Libella Gene Therapy (LGT), specifically utilizing AAV vectors carrying active telomerase (hTERT), to attenuate neuronal senescence. However, the current progress of this research remains undisclosed. In 2022, a novel clinical trial (NCT05040217) was initiated targeting brain-derived neurotrophic factor (BDNF), employing AAV2 as the vector. BDNF exerts regulatory control over neuronal function within the crucial memory circuit of the brain; however, no reports on its outcomes have been published yet.

Currently, the application of CRISPR-Cas9 in AD is primarily in the stage of animal models, encompassing the establishment of more phenotypically relevant models for studying disease pathogenesis and exploring potential treatments [152].

#### Parkinson’s disease

Parkinson's disease (PD) is a heterogeneous disease influenced by multiple factors. To date, more than 100 genes or gene loci associated with susceptibility to PD have been identified (e.g., SNCA, LRRK2, GBA). The majority of cases arise from the combined effect of numerous common or rare genetic mutations. PD is characterized by basal ganglia neuronal dysfunction, where the degeneration of dopamine neurons in the substantia nigra impairs the signal pathway between the substantia nigra and the striatum. The susceptibility genes of PD have complex associations with synaptic, lysosomal, mitochondrial dysfunctions, and immune responses [153]. Based on these revealed potential susceptibility genes, gene therapies targeting these points have been applied to clinical trials. The treatment strategies mainly include protecting neurons, promoting dopamine production, and enhancing the γ-Aminobutyric acid (GABA) signaling pathway.

Neurturin (NTN) and glial cell line-derived neurotrophic factor (GDNF) are neurotrophic factors that confer neuronal protection. The upregulation of NTN and GDNF, promoting neuronal regeneration, represents a therapeutic target for PD. In the phase 2 randomized trial conducted by Marks WJ Jr and colleagues, patients with PD underwent bilateral eputamen injections of AAV2-NTN. The findings revealed no statistically significant difference in the primary endpoints between the treatment group and the control group that received sham surgery [115]. In another clinical trial, researchers demonstrated the feasibility and safety of bilateral stereotactic substantia nigra and putamen injection with AAV2-neurturin (CERE-120) (Class IV evidence) [154]. Clinical trials targeting GDNF include NCT01621581 and NCT04167540, both of which utilize AAV2 as the vector.

Aromatic L-amino acid decarboxylase (AADC) is a crucial enzyme essential for dopamine biosynthesis. Tyrosine hydroxylase (TH) facilitates the conversion of L-tyrosine into the precursor molecule dopamine. The VY-AADC01 trial employed bilateral putamen delivery guided by magnetic resonance imaging (using gadoteridol and AAV2 carrying AADC cDNA). The three dose cohorts exhibited respective increases in enzyme activity of 13% (total dose, ≤ 7.5 × 10^11^vg), 56% (total dose, ≤ 1.5 × 10^12^vg), and 79% (total dose, ≤ 4.7 × 10^12^vg), with enzyme activity assessed via PET [155]. In another trial, a lentivirus vector carrying the gene encoding ADCC, TH, and cyclohydrolase 1 (ProSavin) was employed. Throughout the 12-month follow-up period, no significant adverse effects were observed. A notable improvement in the average UPDRS part III motor scores was evident in the 6th month (mean score 38 [SD 9] vs. 26, *n* = 15, *p* = 0·0001). Furthermore, a sustained enhancement in UPDRS part III motor scores was also noted in the 12th month (38 vs. 27; *n* = 15; *p* = 0·0001) [156]. The clinical trials, namely NCT03562494 (AAV2-DDC, VY-AADC02) and NCT03733496 (AAV2-DDC, VY-AADC01), are currently being conducted.

Glutamic acid decarboxylase (GAD) serves as the rate-limiting enzyme in GABA production, a crucial inhibitory neurotransmitter within the central nervous system. Dysfunction within the GABA signaling pathway has been implicated in PD. The objective of this Phase 2 randomized clinical trial, which enrolled 66 patients with PD, is to evaluate the safety and therapeutic efficacy of AAV2-GAD administration in the subthalamic nucleus. Over a period of 6 months, they initially verified the safety profile and potential treatment benefits associated with AAV2-GAD [157].

Glucosylceramidase, an enzyme localized in the lysosomes and encoded by the GAB1 gene, plays a crucial role in catalyzing the hydrolysis of glucosylceramide into glucose and ceramide. Genetic defects within the GABI gene have been linked to PD [157, 158]. In preclinical models, enhancing the expression of GAB1 may hold therapeutic implications for PD [159]. An ongoing clinical trial (NCT04127578) is utilizing AAV9 carrying the GBA1 gene to evaluate its safety and efficacy against GBA1 deficiency-related PD. Additionally, a clinical trial based on ASO gene therapy BIIB094 (NCT03976349) is currently underway for LRRK2 gene-related PD [160].

#### Amyotrophic lateral sclerosis

Amyotrophic lateral sclerosis (ALS) is a fatal neurodegenerative disease of the central nervous system, which can be divided into sporadic and familial forms, with sporadic cases accounting for approximately 85% of all instances. In addition to the previously acknowledged monogenic inheritance pattern, polygenic inheritance involving multiple genes also significantly contributes to the onset and progression of ALS. Several ALS-related genes have been identified, including penetrant genes such as C9orf72, TARDBP, SOD1, FUS, and genes that confer susceptibility to the disease but do not directly cause it, such as ANG, ATXN2, and DCTN1 [161]. Notably, mutations in the SOD1 gene account for approximately 15% of familial ALS cases [162]. Clinical trials have utilized ASO drugs targeting SOD1 while gene therapies aimed at other causative genes for familial ALS are currently under investigation [163, 164]. Ataxin-2 (ATXN2) is a multifunctional RNA binding protein, primarily serving as a regulatory factor in stress granule assembly. It has been established that the expansion of trinucleotide repeats in ATXN2 can result in spinocerebellar ataxia type 2 (SCA2). Intermediate expansions that do not reach the SCA2 threshold are now recognized as a risk factor for ALS [165]. Additionally, ATXN2 modulates TDP-43 activity and the mutated variant of TDP-43 aggregates into insoluble deposits within brain and spinal neurons. Notably, TDP-43 localizes to atxn2-dependent stress granules, representing a common pathological hallmark [166, 167]. Consequently, gene therapy targeting ATXN2 holds promise not only for addressing ATXN2-related ALS but also for treating other prevalent forms of ALS, particularly sporadic non-familial cases. The ongoing clinical trial (NCT04494256), known as the ALSpire Study, aims to investigate the safety, tolerability, and efficacy of BIIB105 in adult patients with ALS. BIIB105 is an ASO-based drug designed to target ATXN2.

#### Cancer

**Fig. 4:**
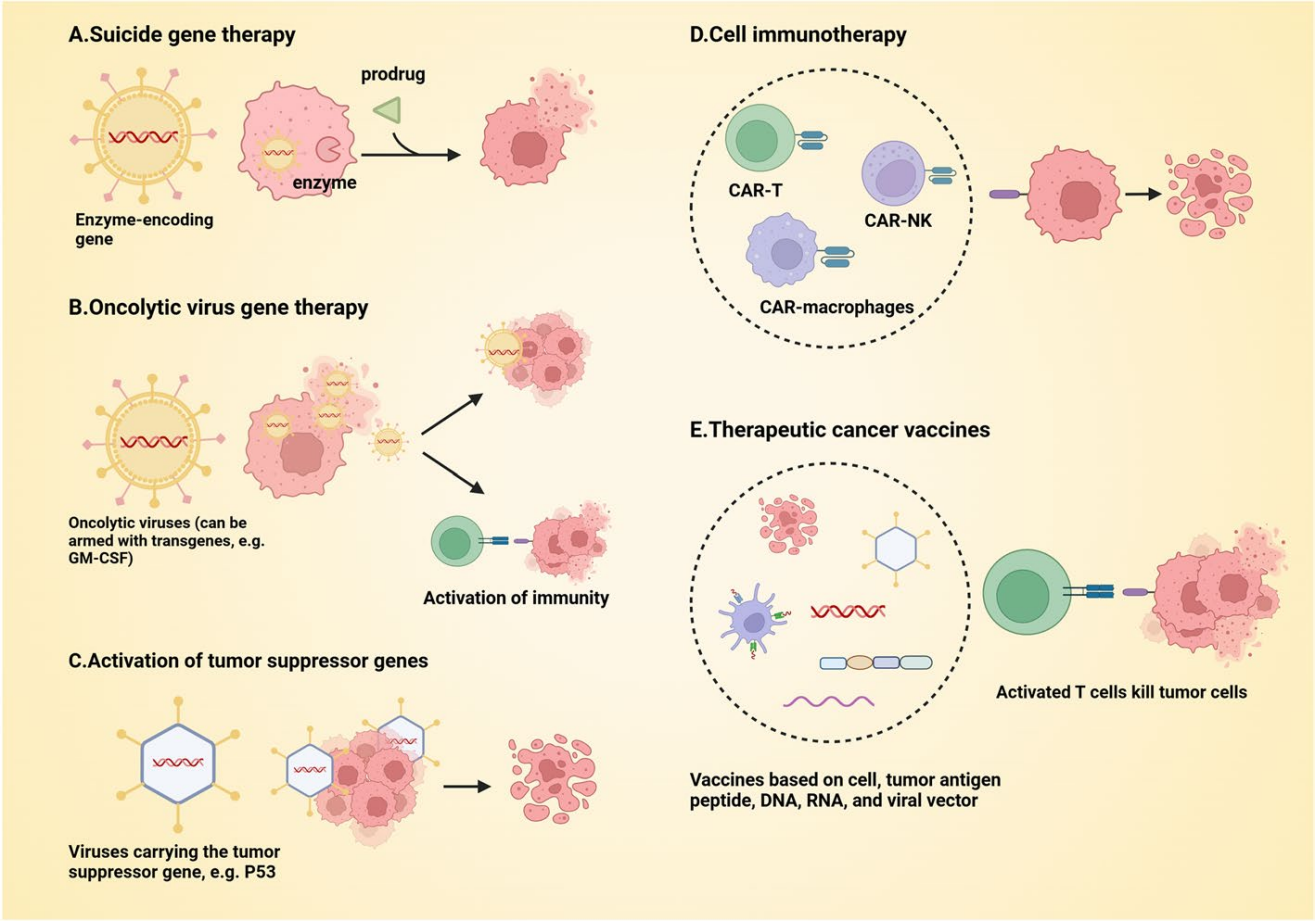
Strategies for tumor gene therapy. GM-CSF: Granulocyte–macrophage colony-stimulating factor; CAR-T: Chimeric antigen receptor T-cell; NK: Natural killer cell. Created with BioRender.com

The underlying molecular mechanisms of cancer are highly intricate and exhibit a significant degree of heterogeneity among diverse individuals. Currently, conventional treatments encompass surgical intervention, radiation therapy, and combined chemotherapy, while gene therapy heralds a new era in the field of cancer treatment. The current strategies for cancer treatment include suicide gene therapy, oncolytic virus gene therapy, activation of tumor suppressor genes, inhibition of oncogene activation, anti-angiogenesis approaches, immunotherapy, and treatments targeting the tumor microenvironment, as well as therapeutic cancer vaccines [168–171](Fig. 4).

Suicide gene therapy employs a carrier to encode a specific enzyme capable of activating the precursor drug, which is subsequently delivered to the tumor cells. The patient then receives the chemotherapy drug in its precursor form, prompting transcription and translation processes within the cell to generate the enzyme required for activation. Consequently, this mechanism effectively eradicates malignant cells [172]. To date, the herpes simplex virus thymidine kinase/ganciclovir system (HSV-TK/GCV) stands as the most extensively utilized suicide gene therapy system [173].

Tumor suppressor genes, including Rb1 (the pioneering anti-cancer gene identified in retinoblastoma), P53, MLH1, APC, MLH1, BRCA, etc., are associated with the development of various cancers due to their inactivation mechanisms involving post-transcriptional modifications [174]. The first approved gene therapy product, Gendicine [175], is a recombinant human P53 Adenovirus Type 5 injection. It introduces the therapeutic gene P53 into target cells and expresses the tumor suppressor gene P53 in these cells, thereby augmenting the body's cancer inhibition mechanism to effectively eradicate tumors. This treatment is approved for head and neck tumors.

Oncolytic viruses, whether natural or modified, have the ability to selectively infect and eradicate tumor cells while sparing normal cells. Among them, adenoviruses and HSV have been extensively investigated. The mechanisms by which oncolytic viruses eliminate tumor cells include direct lysis of infected cells, induction of damage-associated molecular patterns, and pathogen-associated molecular patterns to trigger immune responses in the body [176]. Furthermore, engineered oncolytic viruses can be armed with transgenes such as granulocyte macrophage colony stimulating factor (GM-CSF) [177], interleukin-12 (IL-12) and decorin co-expression [178], CD40L and 4-1BBL co-expression [179], cytosine deaminase [180] and tumor necrosis factor-alpha (TNF alpha) [181]to enhance their efficacy in eradicating tumors. Approved in 2005, Oncorine is the pioneering oncolytic virus product, which is a recombinant adenovirus type 5 injection liquid developed for the treatment of nasopharyngeal cancer [182]. Imlygic, the first oncolytic virus product sanctioned by the U.S. FDA, is an attenuated herpes simplex virus type 1 (HSV1) that has been genetically modified to selectively replicate within tumor cells and express the immune-stimulating protein M-CSF for melanoma therapy [183].

Therapeutic cancer vaccines targeting neoantigens represent a promising avenue for personalized cancer treatment. The majority of cancer patients harbor distinct mutated antigens, which can arise from single nucleotide mutations, insertions, gene fusions, or splicing alterations. These mutations give rise to neoantigenic epitopes that are recognized by T cells and elicit robust anti-tumor immune responses leading to tumor cell eradication. These antigens may exist as singular entities or as combinations of multiple antigenic sites, and they can be shared across different types of tumors [184]. Currently, despite the longstanding proposal of therapeutic cancer vaccines, most of them remain in preclinical or clinical trial stages, indicating a substantial journey ahead before their clinical application can be realized. For instance, Ugur Sahin and colleagues have developed a lipid nanoparticle-based RNA vaccine encoding four frequently observed tumor-associated antigens (NY-ESO-1, MAGE-A3, tyrosinase, TPTE) for melanoma treatment. In this phase I trial, researchers have provided preliminary evidence supporting the feasibility of this vaccine [185].

Since the approval of the first CAR-based cell immunotherapy, Kymriah, in August 2017, a new era has emerged in cancer treatment [186]. Subsequently, the field of gene modification combined with cell immunotherapy has witnessed rapid advancements. Traditional CARs typically comprise four distinct structural regions: 1. A target binding domain, such as a single-chain variable fragment (scFv), responsible for conferring specificity in recognition; 2. A hinge region facilitating flexibility and proper orientation of the CAR construct; 3. A transmembrane domain enabling anchoring of the CAR onto the cell membrane; and 4. Intracellular signaling domains encompassing costimulatory molecules and CD3ζ signaling motifs. Currently, the fifth generation of CAR has emerged, primarily focusing on enhancing intracellular signaling regions to augment immune cell activation and cytotoxicity [187]. Furthermore, diverse iterations of CAR are also under development, including Bivalent CAR, LINK CAR, Stealth CAR, and SNIP CAR, among others [187]. The initial modification of immune cells focused on T cells; however, there has been a recent inclusion of other immune cell types such as NK cells and macrophages in the realm of cellular immunotherapy [188]. The treatment of hematologic malignancies has witnessed significant advancements with the remarkable progress achieved by CAR-T therapy. The currently approved CAR-T products mainly target CD19 and BCMA. According to the analysis of long-term therapeutic effect data, therapies targeting CD19 demonstrate objective remission rates ranging from 44 to 91% in patients with relapsed or refractory B-cell lymphoma or chronic lymphocytic leukemia, and complete remission rates ranging from 28 to 68% (follow-up time ≥ 24 months, time range: 24–123 months). In the case of B-acute lymphoblastic leukemia patients, the initial remission rate ranges from 62 to 86% (follow-up time ≥ 1 year, time range: 1–4.8 years). Limited available data exists for therapies targeting BCMA in patients with relapsed or refractory multiple myeloma; however, overall response rates are reported at levels between 73 and 100%, while complete remission or stringent complete remission rates range from33% to 83% (follow-up time ≥ 1 year, time range:13–48 months) [189]. Currently, CAR-T therapies for solid tumors have not yet received market approval and are still in the clinical trial phase. The targets under development, based on the high expression of antigens in various tumor cell types, encompass HER2, IL-13Rα2, GD2, ROR1, EGFR, CEA, Claudin-18, and MUC1, among others. The identification of specific tumor antigens poses a significant challenge for CAR therapy in the context of solid tumors [190]. Furthermore, efficient delivery and infiltration of CAR into the tumor microenvironment remain formidable obstacles that need to be overcome. Additionally, preventing tumor cell escape and mitigating the immunosuppressive effects exerted by the tumor microenvironment present further challenges [191].

CRISPR technologies offer potential ex vivo therapies for the treatment of human cancers by engineering cellular immunotherapies. For instance, T cells can be modified using CRISPR-Cas9 gRNA-mediated knockout to specifically target PD1 or co-target the T cell receptor, inactivate immunosuppressive factors, and/or integrate CAR elements into the T cell receptor α-chain constant locus for CAR T-cell engineering. These strategies have already progressed to clinical trials. However, in vivo CRISPR therapies are still in pre-clinical stages due to challenges such as efficient and selective delivery and editing [192].

### Endocrine and metabolic diseases

#### Diabetes

Diabetes is traditionally classified into two types, namely Type 1 diabetes (T1D) and Type 2 diabetes (T2D). T1D is characterized by the autoimmune destruction of insulin-producing beta cells in the pancreatic islets, whereas T2D is characterized by cellular resistance to insulin, resulting in relative insulin insufficiency. In recent years, it has been recognized that both T1D and T2D share a common underlying mechanism involving functional impairment of beta cells; however, these mechanisms differ between the two types. The former primarily involves immune-mediated pathways leading to early-stage beta cell destruction, while the latter predominantly involves metabolic pathways with subsequent cell loss occurring at a later stage [193]. Over the past decade, more than 75 genetic signals associated with T1D have been unveiled through GWAS, including variations in HLA alleles [194]. Additionally, over 400 genes linked to T2D, such as AP3S2, GRB14, and TCF7L2, have been identified [195–198]. Currently, gene therapy for diabetes remains at its nascent stage.

The plasticity of pancreatic islet cells has been demonstrated by research findings. Studies conducted by Taka-aki Matsuoka and colleagues have revealed that Pdx1 can induce the differentiation of neurogenin 3 positive endocrine progenitor cells into insulin-positive cells, as well as transdifferentiate glucagon-positive α cell populations into β cells. Furthermore, Mafa can enhance the functionality of Pdx1 in this process, offering promising prospects for diabetes treatment [199]. Subsequently, Xiangwei Xiao and collaborators utilized AAV to deliver Pdx1/MafA, successfully reprogramming α cells into β cells in mice and effectively normalizing blood sugar levels in diabetic model mice [200, 201]. Building upon these advancements, Genprex is currently investigating the therapeutic potential of GPX-002 (AAV-Pdx1/MafA) for treating T1D. As of November 14th, 2023, according to an official website announcement, this trial remains at the preclinical stage [202].

Stem cell therapy has demonstrated advancements in the field of diabetes treatment, exemplified by VX-880. However, the efficacy of stem cell therapy may be hindered by transplant rejection in recipients. VCTX210 integrates stem cell therapy with CRISPR-Cas9 gene editing technology to modify stem cells, thereby disrupting or introducing genes associated with immune rejection (B2M, PD-L1, HLA-E) to achieve immune evasion. Currently, VCTX210 is undergoing phase 1 clinical trials and administration to the first patient has been completed [203]. The other investigational drug GARV-AAV2-A20, currently under research, targets distinct genes [204]. The TNFAIP3 gene encodes the ubiquitin-editing enzyme A20, which modulates immune cell activation by elevating the threshold of NF-κB signals. Overexpression of A20 effectively mitigates inflammatory mediators, thereby safeguarding islet transplants against inflammatory assaults [205]. Currently, these drugs are still in the nascent stages; however, over the course of the next decade, they hold immense potential to revolutionize diabetes treatment.

#### Obesity

Obesity can be classified into polygenic obesity, monogenic obesity, and obesity syndrome based on genetic characteristics, with polygenic obesity being the most prevalent form. The development of polygenic obesity is influenced by a combination of polygenic factors and environmental factors. Rare monogenic obesity primarily involves gene defects related to the leptin-melanocortin pathway, such as the LEP gene encoding leptin, the LEPR gene encoding leptin receptors, and SH2B1. SH2B1 encodes the important SH2B adapter protein 1 that plays a crucial role in mediating leptin-induced signal transduction [206]. Gene therapy for treating obesity is still at an early stage.

In mammals, two distinct types of adipose tissue exist: white adipose tissue (WAT) and brown adipose tissue (BAT). While WAT primarily stores excess fuel, BAT expresses the unique uncoupling protein 1 (UCP1) and possesses the characteristic of energy dissipation. Therefore, activating BAT can increase energy consumption and become a potential target strategy for treating obesity. Chih-Hao Wang and his colleagues utilized the CRISPR-SAM system to activate UCP1 expression in white fat cells, promoting the transformation of WAT into BAT, resulting in significant therapeutic effects in a mouse model. The CRISPR-SAM system has demonstrated its ability to activate endogenous gene expression [207]. Ruhang Tang et al. utilized adeno-associated virus serotype 9 (AAV9) as a vector for delivering the FST gene, and observed its preventive efficacy against high-fat diet-induced obesity [208]. In addition, a recent large-scale exome genome sequencing study involving 645,626 individuals has identified GPR75 as being significantly associated with a lower body mass index. Subsequent investigations using GPR75 gene knockout mice have demonstrated resistance to obesity [209]. Therefore, targeting the downregulation of GPR75 expression may hold promise as a potential therapeutic strategy for combating obesity.

### Other diseases

#### Rheumatoid arthritis

Rheumatoid arthritis (RA) is an autoimmune disease characterized by symmetrical, polyarticular inflammation of small joints. The pathogenesis of RA remains unclear, and it can affect multiple organ systems [210]. Autoimmune reactions and inflammation are the primary mechanisms involved, encompassing various signaling pathways such as JAK-STAT, MAPK, PI3K-AKT, Wnt, Notch, and NF-κB signaling pathways. Additionally, epigenetic modifications also play a crucial role in the onset and progression of RA. Understanding these molecular mechanisms offers a plethora of targets for drug development, encompassing the development of various small molecule inhibitors such as Ruxolitinib, a JAK1/2 inhibitor, VX-702, a p38 MAPK inhibitor, and SHR0302, an exceptionally selective JAK1 inhibitor [211]. In contrast to these small molecule drugs, gene therapy for RA is currently in its nascent preclinical stage. Haobo Han et al. have developed a fluorinated polyamidoamine dendrimer carrier that can be targeted to inflamed joints via intravenous injection for the delivery of miR-23b, a non-coding oligonucleotide that regulates gene expression at the post-transcriptional level. By modulating the NF-κB pathway in proliferating synovium, miR-23b promotes anti-inflammatory and anti-proliferative responses [212]. Additionally, Fang Wang et al. have employed an AAV carrier to co-deliver TNFR-Fc/CTLA4-FasL (AAV-TFCF), which represents another promising gene therapy strategy [213]. The single nucleotide polymorphisms (SNPs) of protein tyrosine phosphatase non-receptor types 2 and 22 (PTPN2/22), specifically PTPN2:rs478582 and PTPN22:rs2476601, may be associated with hyperimmune response and exacerbation of inflammation in RA patients. These SNPs may serve as potential therapeutic targets for managing RA [214]. Moreover, MYC and FOXO1 genes, SNP rs6927172, TNFAIP3, OLIG3 gene, and miR-155 were identified as promising candidate genes for CRISPR-Cas9 therapy [215].

#### Osteoarthritis

Osteoarthritis (OA) is a chronic and progressive disease characterized by the degeneration of joint cartilage, clinically presenting as joint pain and stiffness. Despite its prevalence among more than 500 million individuals, effective treatments for OA are currently lacking [216, 217]. Gene therapy for OA is being actively investigated. Follistatin (FST) functions as a potent inhibitor of muscle growth and an activin binding protein [218]. In their study, Tang et al. employed AAV9-mediated delivery of the FST gene to mitigate inflammatory factors in joint synovial fluid, suggesting that this pathway may hold therapeutic potential for injury-induced OA [208]. The clinical trial NCT05835895 is a phase 1b study designed to investigate the safety, tolerability, and efficacy of GNSC-001 Gene Therapy. GNSC-001 is an AAV-based gene therapy product that carries a gene encoding interleukin-1 receptor antagonist (IL-1Ra). Through intra-articular injection, it transduces cells to produce IL-1Ra, exerting anti-inflammatory and joint protective effects for the treatment of OA. Currently, in the recruitment phase since June 12, 2023, this trial is anticipated to conclude in 2028. In this study [219], Zhao Lan et al. demonstrated that the CRISPR-mediated deletion of nerve growth factor (NGF), matrix metalloproteinase 13 (MMP13), and IL-1 beta genes in a mouse model of post-traumatic OA preserved joint pain management and joint structure. The utilization of CRISPR/Cas9-based gene editing presents a promising therapeutic approach for the treatment of OA.

#### Systemic lupus erythematosus

Systemic lupus erythematosus (SLE) is characterized by autoimmune response-mediated inflammation and organ damage, predominantly affecting young women. Given the pivotal role of B cells in self-antigen recognition during its pathogenesis, gene-engineered immune cell therapy has recently emerged as a promising approach for SLE treatment [220]. In the study conducted by Andreas Mackensen et al., a cohort of 5 SLE patients, who were unresponsive to several immunosuppressive agents, was included. Lentiviral vectors were employed for transducing patient-derived T cells with anti-CD19 CARs. Following lymphocyte depletion pretreatment, the modified T cells were reinfused into the patients at a dose of 1 × 10^6^ CAR T cells per kilogram of body weight. Over a median follow-up period of 8 months, sustained remission without medication was achieved [120]. These findings highlight the potential application of genetically engineered cellular immunotherapy in autoimmune diseases alongside its well-established therapeutic efficacy in cancer treatment. Currently, multiple ongoing clinical trials are investigating potential treatments for recurrent refractory SLE patients. These trials include CAR-NK therapy targeting CD19(NCT06010472), CAR-T therapies targeting both CD19 and BCMA (NCT05858684, NCT05474885), as well as CAR-T therapy specifically targeting CD19(NCT06056921).

#### Future prospects

In the past decade, significant advancements have been achieved in the field of gene therapy, particularly in monogenic diseases and hematological malignancies, with the approval of numerous therapeutic products. Simultaneously, promising prospects have emerged for non-monogenic or complex diseases. By employing comprehensive whole-genome association studies, mendelian randomization analysis, single-cell sequencing of the genome, genetic association research, artificial intelligence machine learning techniques, and other methodologies, we are progressively unraveling the underlying pathogenic mechanisms of complex diseases [221–224]. This approach enables us to effectively identify pathogenic genes implicated in multi-gene disorders and offers novel therapeutic targets for complex disease management. Simultaneously, the progress in genetic engineering technology enables us to intervene in diseases at the DNA or RNA level, supplementing targets that are beyond the reach of conventional drugs. Furthermore, the development of gene therapy products relies on carriers exhibiting high efficiency, robust specificity, low immunogenicity, potent loading capacity, and prolonged duration. The advancement of these carriers is imperative, both viral and nonviral. For instance, in vivo gene therapy extensively employs AAV carriers that can be optimized and modified to acquire exceptional therapeutic properties through approaches like natural isolates, directed evolution, and artificial intelligence-assisted design [225, 226]. For monogenic diseases, the ultimate objective is to achieve long-term or even lifelong cures, as these diseases typically manifest symptoms from birth. Conversely, polygenic and complex diseases tend to emerge later in life. Hence, our focus should not solely be on treating clinical manifestations but rather on early identification of susceptible populations for the purpose of gene prevention and therapy.

## Conclusions

Gene therapy holds great promise as a treatment modality, particularly in the realm of monogenic disorders and tumors. However, with the advancement of our understanding regarding disease pathogenesis, gene therapy is now being applied to address polygenic diseases. Furthermore, given that most polygenic disorders manifest later in life (with symptoms not present at birth), it becomes imperative to identify susceptible individuals for genetic prevention prior to symptom onset. The era of gene therapy is upon us.

### Public summary

1. Significant advancements have been made in the field of gene therapy, leading to the approval of various gene therapy drugs, particularly in monogenic conditions.

2. Building upon the progress achieved in gene therapy for monogenic diseases and cancers, extending its application to polygenic or complex diseases would enable targeting a broader range of patient populations.

## Data Availability

Not applicable.

## References

[1] Cromer MK, Camarena J, Martin RM, Lesch BJ, Vakulskas CA, Bode NM, et al. Gene replacement of α-globin with β-globin restores hemoglobin balance in β-thalassemia-derived hematopoietic stem and progenitor cells. Nat Med. 2021;27(4):677–87. 33737751 10.1038/s41591-021-01284-y PMC8265212

[2] Alshaer W, Zureigat H, Al Karaki A, Al-Kadash A, Gharaibeh L, Hatmal MM, et al. siRNA: Mechanism of action, challenges, and therapeutic approaches. Eur J Pharmacol. 2021;905: 174178. 34044011 10.1016/j.ejphar.2021.174178

[3] Diener C, Keller A, Meese E. Emerging concepts of miRNA therapeutics: from cells to clinic. Trends Genet. 2022;38(6):613–26. 35303998 10.1016/j.tig.2022.02.006

[4] Zabaleta N, Torella L, Weber ND, Gonzalez-Aseguinolaza G. mRNA and gene editing: Late breaking therapies in liver diseases. Hepatology (Baltimore, MD). 2022;76(3):869–87. 35243655 10.1002/hep.32441 PMC9546265

[5] Popovitz J, Sharma R, Hoshyar R, Soo Kim B, Murthy N, Lee K. Gene editing therapeutics based on mRNA delivery. Adv Drug Deliv Rev. 2023;200: 115026. 37516409 10.1016/j.addr.2023.115026

[6] Anderson WF, Blaese RM, Culver K. The ADA human gene therapy clinical protocol: Points to Consider response with clinical protocol, July 6, 1990. Hum Gene Ther. 1990;1(3):331–62. 11642817 10.1089/hum.1990.1.3-331

[7] Blair HA. Valoctocogene Roxaparvovec: First Approval. Drugs. 2022;82(14):1505–10. 36214970 10.1007/s40265-022-01788-y

[8] Heo YA. Etranacogene Dezaparvovec: First Approval. Drugs. 2023;83(4):347–52. 36802324 10.1007/s40265-023-01845-0

[9] Frangoul H, Altshuler D, Cappellini MD, Chen YS, Domm J, Eustace BK, et al. CRISPR-Cas9 Gene Editing for Sickle Cell Disease and β-Thalassemia. N Engl J Med. 2021;384(3):252–60. 33283989 10.1056/NEJMoa2031054

[10] Strauss KA, Farrar MA, Muntoni F, Saito K, Mendell JR, Servais L, et al. Onasemnogene abeparvovec for presymptomatic infants with three copies of SMN2 at risk for spinal muscular atrophy: the Phase III SPR1NT trial. Nat Med. 2022;28(7):1390–7. 35715567 10.1038/s41591-022-01867-3 PMC9205287

[11] Hoy SM. Delandistrogene Moxeparvovec: First Approval. Drugs. 2023;83(14):1323–9. 37566211 10.1007/s40265-023-01929-x

[12] Keam SJ. Eladocagene Exuparvovec: First Approval. Drugs. 2022;82(13):1427–32. 36103022 10.1007/s40265-022-01775-3

[13] Maguire AM, Russell S, Wellman JA, Chung DC, Yu ZF, Tillman A, et al. Efficacy, Safety, and Durability of Voretigene Neparvovec-rzyl in RPE65 Mutation-Associated Inherited Retinal Dystrophy: Results of Phase 1 and 3 Trials. Ophthalmology. 2019;126(9):1273–85. 31443789 10.1016/j.ophtha.2019.06.017

[14] Kaufmann KB, Büning H, Galy A, Schambach A, Grez M. Gene therapy on the move. EMBO Mol Med. 2013;5(11):1642–61. 24106209 10.1002/emmm.201202287 PMC3840483

[15] Gupta D, Bhattacharjee O, Mandal D, Sen MK, Dey D, Dasgupta A, et al. CRISPR-Cas9 system: A new-fangled dawn in gene editing. Life Sci. 2019;232: 116636. 31295471 10.1016/j.lfs.2019.116636

[16] Karimian A, Azizian K, Parsian H, Rafieian S, Shafiei-Irannejad V, Kheyrollah M, et al. CRISPR/Cas9 technology as a potent molecular tool for gene therapy. J Cell Physiol. 2019;234(8):12267–77. 30697727 10.1002/jcp.27972 PMC13484547

[17] Sun N, Zhao H. Transcription activator-like effector nucleases (TALENs): a highly efficient and versatile tool for genome editing. Biotechnol Bioeng. 2013;110(7):1811–21. 23508559 10.1002/bit.24890

[18] Urnov FD, Rebar EJ, Holmes MC, Zhang HS, Gregory PD. Genome editing with engineered zinc finger nucleases. Nat Rev Genet. 2010;11(9):636–46. 20717154 10.1038/nrg2842

[19] Zhao Z, Shang P, Mohanraju P, Geijsen N. Prime editing: advances and therapeutic applications. Trends Biotechnol. 2023;41(8):1000–12. 37002157 10.1016/j.tibtech.2023.03.004

[20] Rees HA, Liu DR. Base editing: precision chemistry on the genome and transcriptome of living cells. Nat Rev Genet. 2018;19(12):770–88. 30323312 10.1038/s41576-018-0059-1 PMC6535181

[21] Durrant MG, Perry NT, Pai JJ, Jangid AR, Athukoralage JS, Hiraizumi M, et al. Bridge RNAs direct programmable recombination of target and donor DNA. Nature. 2024;630(8018):984–93. 38926615 10.1038/s41586-024-07552-4 PMC11208160

[22] Tucci F, Scaramuzza S, Aiuti A, Mortellaro A. Update on clinical Ex Vivo hematopoietic stem cell gene therapy for inherited monogenic diseases. Mole Therapy. 2021;29(2):489–504. 10.1016/j.ymthe.2020.11.020 PMC7854296 33221437

[23] Tucci F, Galimberti S, Naldini L, Valsecchi MG, Aiuti A. A systematic review and meta-analysis of gene therapy with hematopoietic stem and progenitor cells for monogenic disorders. Nat Commun. 2022;13(1):1315. 35288539 10.1038/s41467-022-28762-2 PMC8921234

[24] Blaese RM, Culver KW, Miller AD, Carter CS, Fleisher T, Clerici M, et al. T lymphocyte-directed gene therapy for ADA- SCID: initial trial results after 4 years. Science. 1995;270(5235):475–80. 7570001 10.1126/science.270.5235.475

[25] Song EZ, Milone MC. Pharmacology of Chimeric Antigen Receptor-Modified T Cells. Annu Rev Pharmacol Toxicol. 2021;61:805–29. 33035447 10.1146/annurev-pharmtox-031720-102211

[26] U.S. FOOD & DRUG. CASGEVY. Available from: https://www.fda.gov/vaccines-blood-biologics/casgevy.

[27] U.S. FOOD & DRUG. LYFGENIA. Available from: https://www.fda.gov/vaccines-blood-biologics/lyfgenia.

[28] Brandow AM, Liem RI. Advances in the diagnosis and treatment of sickle cell disease. J Hematol Oncol. 2022;15(1):20. 10.1186/s13045-022-01237-z. 35241123 10.1186/s13045-022-01237-z PMC8895633

[29] Kanter J, Walters MC, Krishnamurti L, Mapara MY, Kwiatkowski JL, Rifkin-Zenenberg S, et al. Biologic and clinical efficacy of lentiglobin for sickle cell disease. N Engl J Med. 2022;386(7):617–28. 34898139 10.1056/NEJMoa2117175

[30] Karagiannis P, Takahashi K, Saito M, Yoshida Y, Okita K, Watanabe A, et al. Induced pluripotent stem cells and their use in human models of disease and development. Physiol Rev. 2019;99(1):79–114. 30328784 10.1152/physrev.00039.2017

[31] Orqueda AJ, Giménez CA, Pereyra-Bonnet F. iPSCs: A Minireview from Bench to Bed, including Organoids and the CRISPR System. Stem cells international. 2016;2016:5934782. 26880972 10.1155/2016/5934782 PMC4736429

[32] Tang LV, Tao Y, Feng Y, Ma J, Lin W, Zhang Y, et al. Gene editing of human iPSCs rescues thrombophilia in hereditary antithrombin deficiency in mice. Sci Transl Med. 2022;14(673):eabq3202. 36449603 10.1126/scitranslmed.abq3202

[33] Nathwani AC, Tuddenham EG, Rangarajan S, Rosales C, McIntosh J, Linch DC, et al. Adenovirus-associated virus vector-mediated gene transfer in hemophilia B. N Engl J Med. 2011;365(25):2357–65. 22149959 10.1056/NEJMoa1108046 PMC3265081

[34] Park YM, Woo S, Lee GT, Ko JY, Lee Y, Zhao ZS, et al. Safety and efficacy of adeno-associated viral vector-mediated insulin gene transfer via portal vein to the livers of streptozotocin-induced diabetic Sprague-Dawley rats. J Gene Med. 2005;7(5):621–9. 15651056 10.1002/jgm.708

[35] Tasfaout H, Lionello VM, Kretz C, Koebel P, Messaddeq N, Bitz D, et al. Single Intramuscular Injection of AAV-shRNA Reduces DNM2 and Prevents Myotubular Myopathy in Mice. Mole Therapy. 2018;26(4):1082–92. 10.1016/j.ymthe.2018.02.008 PMC6080128 29506908

[36] Watanabe M, Nishikawaji Y, Kawakami H, Kosai KI. Adenovirus Biology, Recombinant Adenovirus, and Adenovirus Usage in Gene Therapy. Viruses. 2021;13(12):2502. 10.3390/v13122502. 34960772 10.3390/v13122502 PMC8706629

[37] Trivedi PD, Byrne BJ, Corti M. Evolving Horizons: Adenovirus Vectors’ Timeless Influence on Cancer, Gene Therapy and Vaccines. Viruses. 2023;15(12):2378. 10.3390/v15122378. 38140619 10.3390/v15122378 PMC10747483

[38] Wang D, Tai PWL, Gao G. Adeno-associated virus vector as a platform for gene therapy delivery. Nat Rev Drug Discovery. 2019;18(5):358–78. 30710128 10.1038/s41573-019-0012-9 PMC6927556

[39] Yakovlev IA, Emelin AM, Slesarenko YS, Limaev IS, Vetrova IA, Belikova LD, et al. Dual Adeno-Associated Virus 9 with Codon-Optimized DYSF Gene Promotes In Vivo Muscle Regeneration and May Decrease Inflammatory Response in Limb Girdle Muscular Dystrophy Type R2. Int J Mol Sci. 2023;24(17):13551. 10.3390/ijms241713551. 37686363 10.3390/ijms241713551 PMC10488094

[40] Li X, Wei X, Lin J, Ou L. A versatile toolkit for overcoming AAV immunity. Front Immunol. 2022;13: 991832. 36119036 10.3389/fimmu.2022.991832 PMC9479010

[41] Bulcha JT, Wang Y, Ma H, Tai PWL, Gao G. Viral vector platforms within the gene therapy landscape. Signal Transduct Target Ther. 2021;6(1):53. 10.1038/s41392-021-00487-6. 33558455 10.1038/s41392-021-00487-6 PMC7868676

[42] Frampton AR Jr, Goins WF, Nakano K, Burton EA, Glorioso JC. HSV trafficking and development of gene therapy vectors with applications in the nervous system. Gene Ther. 2005;12(11):891–901. 15908995 10.1038/sj.gt.3302545

[43] Guide SV, Gonzalez ME, Bağcı IS, Agostini B, Chen H, Feeney G, et al. Trial of Beremagene Geperpavec (B-VEC) for Dystrophic Epidermolysis Bullosa. N Engl J Med. 2022;387(24):2211–9. 36516090 10.1056/NEJMoa2206663

[44] Knight S, Collins M, Takeuchi Y. Insertional mutagenesis by retroviral vectors: current concepts and methods of analysis. Curr Gene Ther. 2013;13(3):211–27. 23590635 10.2174/1566523211313030006

[45] Shirley JL, de Jong YP, Terhorst C, Herzog RW. Immune Responses to Viral Gene Therapy Vectors. Molecular therapy : the journal of the American Society of Gene Therapy. 2020;28(3):709–22. 31968213 10.1016/j.ymthe.2020.01.001 PMC7054714

[46] El Andari J, Grimm D. Production, Processing, and Characterization of Synthetic AAV Gene Therapy Vectors. Biotechnol J. 2021;16(1): e2000025. 32975881 10.1002/biot.202000025

[47] Ren S, Wang M, Wang C, Wang Y, Sun C, Zeng Z, Cui H, Zhao X. Application of Non-Viral Vectors in Drug Delivery and Gene Therapy. Polymers (Basel). 2021;13(19):3307. 10.3390/polym13193307. 34641123 10.3390/polym13193307 PMC8512075

[48] Wang C, Pan C, Yong H, Wang F, Bo T, Zhao Y, Ma B, He W, Li M. Emerging non-viral vectors for gene delivery. J Nanobiotechnol. 2023;21(1):272. 10.1186/s12951-023-02044-5. 10.1186/s12951-023-02044-5 PMC10433663 37592351

[49] Kreitz J, Friedrich MJ, Guru A, Lash B, Saito M, Macrae RK, et al. Programmable protein delivery with a bacterial contractile injection system. Nature. 2023;616(7956):357–64. 36991127 10.1038/s41586-023-05870-7 PMC10097599

[50] van Rheenen W, Peyrot WJ, Schork AJ, Lee SH, Wray NR. Genetic correlations of polygenic disease traits: from theory to practice. Nat Rev Genet. 2019;20(10):567–81. 31171865 10.1038/s41576-019-0137-z

[51] Crouch DJM, Bodmer WF. Polygenic inheritance, GWAS, polygenic risk scores, and the search for functional variants. Proc Natl Acad Sci. 2020;117(32):18924–33. 32753378 10.1073/pnas.2005634117 PMC7431089

[52] Oliynyk RT. Future Preventive Gene Therapy of Polygenic Diseases from a Population Genetics Perspective. Int J Mol Sci. 2019;20(20):5013. 10.3390/ijms20205013. 31658652 10.3390/ijms20205013 PMC6834143

[53] Kukuła K, Urbanowicz A, Kłopotowski M, Dąbrowski M, Pręgowski J, Kądziela J, et al. Long-term follow-up and safety assessment of angiogenic gene therapy trial VIF-CAD: Transcatheter intramyocardial administration of a bicistronic plasmid expressing VEGF-A165/bFGF cDNA for the treatment of refractory coronary artery disease. Am Heart J. 2019;215:78–82. 31288177 10.1016/j.ahj.2019.06.009

[54] Symes JF, Losordo DW, Vale PR, Lathi KG, Esakof DD, Mayskiy M, et al. Gene therapy with vascular endothelial growth factor for inoperable coronary artery disease. Ann Thorac Surg. 1999;68(3):830–6; discussion 6–7. 10.1016/s0003-4975(99)00807-3 10509970

[55] Kołsut P, Małecki M, Zelazny P, Teresińska A, Firek B, Janik P, et al. Gene therapy of coronary artery disease with phvegf165–early outcome. Kardiol Pol. 2003;59(11):373–84. 14668888

[56] Tio RA, Tan ES, Jessurun GA, Veeger N, Jager PL, Slart RH, et al. PET for evaluation of differential myocardial perfusion dynamics after VEGF gene therapy and laser therapy in end-stage coronary artery disease. Journal of nuclear medicine : official publication, Society of Nuclear Medicine. 2004;45(9):1437–43. 15347709

[57] Rosengart TK, Bishawi MM, Halbreiner MS, Fakhoury M, Finnin E, Hollmann C, et al. Long-term follow-up assessment of a phase 1 trial of angiogenic gene therapy using direct intramyocardial administration of an adenoviral vector expressing the VEGF121 cDNA for the treatment of diffuse coronary artery disease. Hum Gene Ther. 2013;24(2):203–8. 23137122 10.1089/hum.2012.137 PMC3581022

[58] Rosengart TK, Lee LY, Patel SR, Sanborn TA, Parikh M, Bergman GW, et al. Angiogenesis gene therapy: phase I assessment of direct intramyocardial administration of an adenovirus vector expressing VEGF121 cDNA to individuals with clinically significant severe coronary artery disease. Circulation. 1999;100(5):468–74. 10430759 10.1161/01.CIR.100.5.468

[59] Lathi KG, Vale PR, Losordo DW, Cespedes RM, Symes JF, Esakof DD, et al. Gene therapy with vascular endothelial growth factor for inoperable coronary artery disease: anesthetic management and results. Anesth Analg. 2001;92(1):19–25. 11133594 10.1097/00000539-200101000-00005

[60] Kastrup J, Jørgensen E, Fuchs S, Nikol S, Bøtker HE, Gyöngyösi M, et al. A randomised, double-blind, placebo-controlled, multicentre study of the safety and efficacy of BIOBYPASS (AdGVVEGF121.10NH) gene therapy in patients with refractory advanced coronary artery disease: the NOVA trial. EuroIntervention. 2011;6(7):813–8. 21252014 10.4244/EIJV6I7A140

[61] Hassinen I, Kivelä A, Hedman A, Saraste A, Knuuti J, Hartikainen J, et al. Intramyocardial Gene Therapy Directed to Hibernating Heart Muscle Using a Combination of Electromechanical Mapping and Positron Emission Tomography. Hum Gene Ther. 2016;27(10):830–4. 27553362 10.1089/hum.2016.131

[62] Sarkar N, Rück A, Källner G, S YH, Blomberg P, Islam KB, et al. Effects of intramyocardial injection of phVEGF-A165 as sole therapy in patients with refractory coronary artery disease--12-month follow-up: angiogenic gene therapy. J Int Med. 2001;250(5):373–81. 10.1046/j.1365-2796.2001.00905.x 11887971

[63] Stewart DJ, Hilton JD, Arnold JM, Gregoire J, Rivard A, Archer SL, et al. Angiogenic gene therapy in patients with nonrevascularizable ischemic heart disease: a phase 2 randomized, controlled trial of AdVEGF(121) (AdVEGF121) versus maximum medical treatment. Gene Ther. 2006;13(21):1503–11. 16791287 10.1038/sj.gt.3302802

[64] Yang ZJ, Zhang YR, Chen B, Zhang SL, Jia EZ, Wang LS, et al. Phase I clinical trial on intracoronary administration of Ad-hHGF treating severe coronary artery disease. Mol Biol Rep. 2009;36(6):1323–9. 18649012 10.1007/s11033-008-9315-3

[65] Meng H, Chen B, Tao Z, Xu Z, Wang L, Weizhu J, et al. Safety and Efficacy of Adenovirus Carrying Hepatocyte Growth Factor Gene by Percutaneous Endocardial Injection for Treating Post-infarct Heart Failure: A Phase IIa Clinical Trial. Curr Gene Ther. 2018;18(2):125–30. 29618307 10.2174/1566523218666180404162209

[66] Losordo DW, Vale PR, Symes JF, Dunnington CH, Esakof DD, Maysky M, et al. Gene therapy for myocardial angiogenesis: initial clinical results with direct myocardial injection of phVEGF165 as sole therapy for myocardial ischemia. Circulation. 1998;98(25):2800–4. 9860779 10.1161/01.CIR.98.25.2800

[67] Vale PR, Losordo DW, Milliken CE, Maysky M, Esakof DD, Symes JF, et al. Left ventricular electromechanical mapping to assess efficacy of phVEGF(165) gene transfer for therapeutic angiogenesis in chronic myocardial ischemia. Circulation. 2000;102(9):965–74. 10961959 10.1161/01.CIR.102.9.965

[68] Hedman M, Hartikainen J, Syvänne M, Stjernvall J, Hedman A, Kivelä A, et al. Safety and feasibility of catheter-based local intracoronary vascular endothelial growth factor gene transfer in the prevention of postangioplasty and in-stent restenosis and in the treatment of chronic myocardial ischemia: phase II results of the Kuopio Angiogenesis Trial (KAT). Circulation. 2003;107(21):2677–83. 12742981 10.1161/01.CIR.0000070540.80780.92

[69] Kastrup J, Jørgensen E, Rück A, Tägil K, Glogar D, Ruzyllo W, et al. Direct intramyocardial plasmid vascular endothelial growth factor-A165 gene therapy in patients with stable severe angina pectoris A randomized double-blind placebo-controlled study: the Euroinject One trial. J Am Coll Cardiol. 2005;45(7):982–8. 15808751 10.1016/j.jacc.2004.12.068

[70] Stewart DJ, Kutryk MJB, Fitchett D, Freeman M, Camack N, Su Y, et al. VEGF Gene Therapy Fails to Improve Perfusion of Ischemic Myocardium in Patients With Advanced Coronary Disease: Results of the NORTHERN Trial. Mol Ther. 2009;17(6):1109–15. 19352324 10.1038/mt.2009.70 PMC2835194

[71] Ripa RS, Wang Y, Jørgensen E, Johnsen HE, Hesse B, Kastrup J. Intramyocardial injection of vascular endothelial growth factor-A165 plasmid followed by granulocyte-colony stimulating factor to induce angiogenesis in patients with severe chronic ischaemic heart disease. Eur Heart J. 2006;27(15):1785–92. 16825290 10.1093/eurheartj/ehl117

[72] Grines CL, Watkins MW, Helmer G, Penny W, Brinker J, Marmur JD, et al. Angiogenic Gene Therapy (AGENT) trial in patients with stable angina pectoris. Circulation. 2002;105(11):1291–7. 11901038 10.1161/hc1102.105595

[73] Grines CL, Watkins MW, Mahmarian JJ, Iskandrian AE, Rade JJ, Marrott P, et al. A randomized, double-blind, placebo-controlled trial of Ad5FGF-4 gene therapy and its effect on myocardial perfusion in patients with stable angina. J Am Coll Cardiol. 2003;42(8):1339–47. 14563572 10.1016/S0735-1097(03)00988-4

[74] Kim HJ, Jang SY, Park JI, Byun J, Kim DI, Do YS, et al. Vascular endothelial growth factor-induced angiogenic gene therapy in patients with peripheral artery disease. Exp Mol Med. 2004;36(4):336–44. 15365252 10.1038/emm.2004.44

[75] Niebuhr A, Henry T, Goldman J, Baumgartner I, van Belle E, Gerss J, et al. Long-term safety of intramuscular gene transfer of non-viral FGF1 for peripheral artery disease. Gene Ther. 2012;19(3):264–70. 21716303 10.1038/gt.2011.85

[76] Gu Y, Cui S, Liu C, Zhao J, Li M, Li Y, et al. pUDK-HGF Gene Therapy to Relieve CLI Rest Pain and Ulcer: A Phase II, Double-Blind. Randomized Placebo-Controlled Trial Human gene therapy. 2021;32(15–16):839–49. 33726499 10.1089/hum.2020.290

[77] Barć P, Antkiewicz M, Śliwa B, Frączkowska K, Guziński M, Dawiskiba T, et al. Double VEGF/HGF Gene Therapy in Critical Limb Ischemia Complicated by Diabetes Mellitus. J Cardiovasc Transl Res. 2021;14(3):409–15. 32875492 10.1007/s12265-020-10066-9 PMC8219552

[78] Rajagopalan S, Mohler ER 3rd, Lederman RJ, Mendelsohn FO, Saucedo JF, Goldman CK, et al. Regional angiogenesis with vascular endothelial growth factor in peripheral arterial disease: a phase II randomized, double-blind, controlled study of adenoviral delivery of vascular endothelial growth factor 121 in patients with disabling intermittent claudication. Circulation. 2003;108(16):1933–8. 14504183 10.1161/01.CIR.0000093398.16124.29

[79] Makino H, Aoki M, Hashiya N, Yamasaki K, Azuma J, Sawa Y, et al. Long-term follow-up evaluation of results from clinical trial using hepatocyte growth factor gene to treat severe peripheral arterial disease. Arterioscler Thromb Vasc Biol. 2012;32(10):2503–9. 22904270 10.1161/ATVBAHA.111.244632

[80] Kusumanto YH, van Weel V, Mulder NH, Smit AJ, van den Dungen JJ, Hooymans JM, et al. Treatment with intramuscular vascular endothelial growth factor gene compared with placebo for patients with diabetes mellitus and critical limb ischemia: a double-blind randomized trial. Hum Gene Ther. 2006;17(6):683–91. 16776576 10.1089/hum.2006.17.683

[81] Shishehbor MH, Rundback J, Bunte M, Hammad TA, Miller L, Patel PD, et al. SDF-1 plasmid treatment for patients with peripheral artery disease (STOP-PAD): Randomized, double-blind, placebo-controlled clinical trial. Vascular medicine (London, England). 2019;24(3):200–7. 30786835 10.1177/1358863X18817610

[82] Deev R, Plaksa I, Bozo I, Mzhavanadze N, Suchkov I, Chervyakov Y, et al. Results of 5-year follow-up study in patients with peripheral artery disease treated with PL-VEGF165 for intermittent claudication. Ther Adv Cardiovasc Dis. 2018;12(9):237–46. 29996720 10.1177/1753944718786926 PMC6116753

[83] Rajagopalan S, Shah M, Luciano A, Crystal R, Nabel EG. Adenovirus-mediated gene transfer of VEGF(121) improves lower-extremity endothelial function and flow reserve. Circulation. 2001;104(7):753–5. 11502697 10.1161/hc3201.095192

[84] Mohler ER 3rd, Rajagopalan S, Olin JW, Trachtenberg JD, Rasmussen H, Pak R, et al. Adenoviral-mediated gene transfer of vascular endothelial growth factor in critical limb ischemia: safety results from a phase I trial. Vascular medicine (London, England). 2003;8(1):9–13. 12866606 10.1191/1358863x03vm460oa

[85] Gu Y, Cui S, Wang Q, Liu C, Jin B, Guo W, et al. A Randomized, Double-Blind, Placebo-Controlled Phase II Study of Hepatocyte Growth Factor in the Treatment of Critical Limb Ischemia. Molecular therapy : the journal of the American Society of Gene Therapy. 2019;27(12):2158–65. 31805256 10.1016/j.ymthe.2019.10.017 PMC6904746

[86] Yonemitsu Y, Matsumoto T, Itoh H, Okazaki J, Uchiyama M, Yoshida K, et al. DVC1-0101 to treat peripheral arterial disease: a Phase I/IIa open-label dose-escalation clinical trial. Molecular therapy : the journal of the American Society of Gene Therapy. 2013;21(3):707–14. 23319060 10.1038/mt.2012.279 PMC3589164

[87] Cui S, Guo L, Li X, Gu Y, Fu J, Dong L, et al. Clinical Safety and Preliminary Efficacy of Plasmid pUDK-HGF Expressing Human Hepatocyte Growth Factor (HGF) in Patients with Critical Limb Ischemia. European journal of vascular and endovascular surgery : the official journal of the European Society for Vascular Surgery. 2015;50(4):494–501. 26122834 10.1016/j.ejvs.2015.05.007

[88] Henry TD, Hirsch AT, Goldman J, Wang YL, Lips DL, McMillan WD, et al. Safety of a non-viral plasmid-encoding dual isoforms of hepatocyte growth factor in critical limb ischemia patients: a phase I study. Gene Ther. 2011;18(8):788–94. 21430785 10.1038/gt.2011.21

[89] Gu Y, Zhang J, Guo L, Cui S, Li X, Ding D, et al. A phase I clinical study of naked DNA expressing two isoforms of hepatocyte growth factor to treat patients with critical limb ischemia. J Gene Med. 2011;13(11):602–10. 22015632 10.1002/jgm.1614

[90] Rajagopalan S, Trachtenberg J, Mohler E, Olin J, McBride S, Pak R, et al. Phase I study of direct administration of a replication deficient adenovirus vector containing the vascular endothelial growth factor cDNA (CI-1023) to patients with claudication. Am J Cardiol. 2002;90(5):512–6. 12208412 10.1016/S0002-9149(02)02524-9

[91] Lyon AR, Babalis D, Morley-Smith AC, Hedger M, Suarez Barrientos A, Foldes G, et al. Investigation of the safety and feasibility of AAV1/SERCA2a gene transfer in patients with chronic heart failure supported with a left ventricular assist device - the SERCA-LVAD TRIAL. Gene Ther. 2020;27(12):579–90. 32669717 10.1038/s41434-020-0171-7 PMC7744277

[92] Chung ES, Miller L, Patel AN, Anderson RD, Mendelsohn FO, Traverse J, et al. Changes in ventricular remodelling and clinical status during the year following a single administration of stromal cell-derived factor-1 non-viral gene therapy in chronic ischaemic heart failure patients: the STOP-HF randomized Phase II trial. Eur Heart J. 2015;36(33):2228–38. 26056125 10.1093/eurheartj/ehv254 PMC4554960

[93] Jessup M, Greenberg B, Mancini D, Cappola T, Pauly DF, Jaski B, et al. Calcium Upregulation by Percutaneous Administration of Gene Therapy in Cardiac Disease (CUPID): a phase 2 trial of intracoronary gene therapy of sarcoplasmic reticulum Ca2+-ATPase in patients with advanced heart failure. Circulation. 2011;124(3):304–13. 21709064 10.1161/CIRCULATIONAHA.111.022889 PMC5843948

[94] Zsebo K, Yaroshinsky A, Rudy JJ, Wagner K, Greenberg B, Jessup M, et al. Long-term effects of AAV1/SERCA2a gene transfer in patients with severe heart failure: analysis of recurrent cardiovascular events and mortality. Circ Res. 2014;114(1):101–8. 24065463 10.1161/CIRCRESAHA.113.302421

[95] Greenberg B, Yaroshinsky A, Zsebo KM, Butler J, Felker GM, Voors AA, et al. Design of a phase 2b trial of intracoronary administration of AAV1/SERCA2a in patients with advanced heart failure: the CUPID 2 trial (calcium up-regulation by percutaneous administration of gene therapy in cardiac disease phase 2b). JACC Heart failure. 2014;2(1):84–92. 24622121 10.1016/j.jchf.2013.09.008

[96] Hammond HK, Penny WF, Traverse JH, Henry TD, Watkins MW, Yancy CW, et al. Intracoronary Gene Transfer of Adenylyl Cyclase 6 in Patients With Heart Failure: A Randomized Clinical Trial. JAMA cardiology. 2016;1(2):163–71. 27437887 10.1001/jamacardio.2016.0008 PMC5535743

[97] Hulot JS, Salem JE, Redheuil A, Collet JP, Varnous S, Jourdain P, et al. Effect of intracoronary administration of AAV1/SERCA2a on ventricular remodelling in patients with advanced systolic heart failure: results from the AGENT-HF randomized phase 2 trial. Eur J Heart Fail. 2017;19(11):1534–41. 28393439 10.1002/ejhf.826

[98] Ray KK, Wright RS, Kallend D, Koenig W, Leiter LA, Raal FJ, et al. Two Phase 3 Trials of Inclisiran in Patients with Elevated LDL Cholesterol. N Engl J Med. 2020;382(16):1507–19. 32187462 10.1056/NEJMoa1912387

[99] Raal FJ, Kallend D, Ray KK, Turner T, Koenig W, Wright RS, et al. Inclisiran for the Treatment of Heterozygous Familial Hypercholesterolemia. N Engl J Med. 2020;382(16):1520–30. 32197277 10.1056/NEJMoa1913805

[100] Ray KK, Landmesser U, Leiter LA, Kallend D, Dufour R, Karakas M, et al. Inclisiran in Patients at High Cardiovascular Risk with Elevated LDL Cholesterol. N Engl J Med. 2017;376(15):1430–40. 28306389 10.1056/NEJMoa1615758

[101] Ray KK, Troquay RPT, Visseren FLJ, Leiter LA, Scott Wright R, Vikarunnessa S, et al. Long-term efficacy and safety of inclisiran in patients with high cardiovascular risk and elevated LDL cholesterol (ORION-3): results from the 4-year open-label extension of the ORION-1 trial. Lancet Diabetes Endocrinol. 2023;11(2):109–19. 36620965 10.1016/S2213-8587(22)00353-9

[102] Raal F, Durst R, Bi R, Talloczy Z, Maheux P, Lesogor A, et al. Efficacy, Safety, and Tolerability of Inclisiran in Patients With Homozygous Familial Hypercholesterolemia: Results From the ORION-5 Randomized Clinical Trial. Circulation. 2024;149(5):354–62. 37850379 10.1161/CIRCULATIONAHA.122.063460 PMC10815002

[103] O’Donoghue ML, Rosenson RS, Gencer B, López JAG, Lepor NE, Baum SJ, et al. Small Interfering RNA to Reduce Lipoprotein(a) in Cardiovascular Disease. N Engl J Med. 2022;387(20):1855–64. 36342163 10.1056/NEJMoa2211023

[104] Yeang C, Karwatowska-Prokopczuk E, Su F, Dinh B, Xia S, Witztum JL, et al. Effect of Pelacarsen on Lipoprotein(a) Cholesterol and Corrected Low-Density Lipoprotein Cholesterol. J Am Coll Cardiol. 2022;79(11):1035–46. 35300814 10.1016/j.jacc.2021.12.032 PMC8972555

[105] Gaudet D, Karwatowska-Prokopczuk E, Baum SJ, Hurh E, Kingsbury J, Bartlett VJ, et al. Vupanorsen, an N-acetyl galactosamine-conjugated antisense drug to ANGPTL3 mRNA, lowers triglycerides and atherogenic lipoproteins in patients with diabetes, hepatic steatosis, and hypertriglyceridaemia. Eur Heart J. 2020;41(40):3936–45. 32860031 10.1093/eurheartj/ehaa689 PMC7750927

[106] Bergmark BA, Marston NA, Bramson CR, Curto M, Ramos V, Jevne A, et al. Effect of Vupanorsen on Non-High-Density Lipoprotein Cholesterol Levels in Statin-Treated Patients With Elevated Cholesterol: TRANSLATE-TIMI 70. Circulation. 2022;145(18):1377–86. 35369705 10.1161/CIRCULATIONAHA.122.059266 PMC9047643

[107] Witztum JL, Gaudet D, Freedman SD, Alexander VJ, Digenio A, Williams KR, et al. Volanesorsen and Triglyceride Levels in Familial Chylomicronemia Syndrome. N Engl J Med. 2019;381(6):531–42. 31390500 10.1056/NEJMoa1715944

[108] Gouni-Berthold I, Alexander VJ, Yang Q, Hurh E, Steinhagen-Thiessen E, Moriarty PM, et al. Efficacy and safety of volanesorsen in patients with multifactorial chylomicronaemia (COMPASS): a multicentre, double-blind, randomised, placebo-controlled, phase 3 trial. Lancet Diabetes Endocrinol. 2021;9(5):264–75. 33798466 10.1016/S2213-8587(21)00046-2

[109] Bakris GL, Saxena M, Gupta A, Chalhoub F, Lee J, Stiglitz D, et al. RNA Interference With Zilebesiran for Mild to Moderate Hypertension: The KARDIA-1 Randomized Clinical Trial. JAMA. 2024;331(9):740–9. 38363577 10.1001/jama.2024.0728 PMC10873804

[110] Nakagami H, Ishihama T, Daikyoji Y, Sasakura C, Yamada E, Morishita R. Brief report on a phase I/IIa study to assess the safety, tolerability, and immune response of AGMG0201 in patients with essential hypertension. Hypertens Res. 2021;45(1):61–5. 34657138 10.1038/s41440-021-00755-6 PMC8668431

[111] Rafii MS, Tuszynski MH, Thomas RG, Barba D, Brewer JB, Rissman RA, et al. Adeno-Associated Viral Vector (Serotype 2)-Nerve Growth Factor for Patients With Alzheimer Disease: A Randomized Clinical Trial. JAMA Neurol. 2018;75(7):834–41. 29582053 10.1001/jamaneurol.2018.0233 PMC5885277

[112] Rafii MS, Baumann TL, Bakay RA, Ostrove JM, Siffert J, Fleisher AS, et al. A phase1 study of stereotactic gene delivery of AAV2-NGF for Alzheimer’s disease. Alzheimer’s Dementia. 2014;10(5):571–81. 24411134 10.1016/j.jalz.2013.09.004

[113] Kaplitt MG, Feigin A, Tang C, Fitzsimons HL, Mattis P, Lawlor PA, et al. Safety and tolerability of gene therapy with an adeno-associated virus (AAV) borne GAD gene for Parkinson’s disease: an open label, phase I trial. Lancet (London, England). 2007;369(9579):2097–105. 17586305 10.1016/S0140-6736(07)60982-9

[114] Marks WJ Jr, Ostrem JL, Verhagen L, Starr PA, Larson PS, Bakay RA, et al. Safety and tolerability of intraputaminal delivery of CERE-120 (adeno-associated virus serotype 2-neurturin) to patients with idiopathic Parkinson’s disease: an open-label, phase I trial. The Lancet Neurology. 2008;7(5):400–8. 18387850 10.1016/S1474-4422(08)70065-6

[115] Marks WJ, Bartus RT, Siffert J, Davis CS, Lozano A, Boulis N, et al. Gene delivery of AAV2-neurturin for Parkinson’s disease: a double-blind, randomised, controlled trial. The Lancet Neurology. 2010;9(12):1164–72. 20970382 10.1016/S1474-4422(10)70254-4

[116] Muramatsu S, Fujimoto K, Kato S, Mizukami H, Asari S, Ikeguchi K, et al. A phase I study of aromatic L-amino acid decarboxylase gene therapy for Parkinson’s disease. Molecular therapy : the journal of the American Society of Gene Therapy. 2010;18(9):1731–5. 20606642 10.1038/mt.2010.135 PMC2956925

[117] Warren Olanow C, Bartus RT, Baumann TL, Factor S, Boulis N, Stacy M, et al. Gene delivery of neurturin to putamen and substantia nigra in Parkinson disease: A double-blind, randomized, controlled trial. Ann Neurol. 2015;78(2):248–57. 26061140 10.1002/ana.24436

[118] Niethammer M, Tang CC, LeWitt PA, Rezai AR, Leehey MA, Ojemann SG, et al. Long-term follow-up of a randomized AAV2-GAD gene therapy trial for Parkinson’s disease. JCI insight. 2017;2(7): e90133. 28405611 10.1172/jci.insight.90133 PMC5374069

[119] Mittermeyer G, Christine CW, Rosenbluth KH, Baker SL, Starr P, Larson P, et al. Long-term evaluation of a phase 1 study of AADC gene therapy for Parkinson’s disease. Hum Gene Ther. 2012;23(4):377–81. 22424171 10.1089/hum.2011.220 PMC4530392

[120] Mackensen A, Müller F, Mougiakakos D, Böltz S, Wilhelm A, Aigner M, et al. Anti-CD19 CAR T cell therapy for refractory systemic lupus erythematosus. Nat Med. 2022;28(10):2124–32. 36109639 10.1038/s41591-022-02017-5

[121] Losordo DW, Vale PR, Symes JF, Dunnington CH, Esakof DD, Maysky M, et al. Gene Therapy for Myocardial Angiogenesis. Circulation. 1998;98(25):2800–4. 9860779 10.1161/01.CIR.98.25.2800

[122] Hartikainen J, Hassinen I, Hedman A, Kivelä A, Saraste A, Knuuti J, et al. Adenoviral intramyocardial VEGF-DΔNΔC gene transfer increases myocardial perfusion reserve in refractory angina patients: a phase I/IIa study with 1-year follow-up. Eur Heart J. 2017;38(33):2547–55. 28903476 10.1093/eurheartj/ehx352 PMC5837555

[123] Kaski JC, Consuegra-Sanchez L. Evaluation of ASPIRE trial: a Phase III pivotal registration trial, using intracoronary administration of Generx (Ad5FGF4) to treat patients with recurrent angina pectoris. Expert Opin Biol Ther. 2013;13(12):1749–53. 23957658 10.1517/14712598.2013.827656

[124] Ripa RS. Intramyocardial injection of vascular endothelial growth factor-A165 plasmid followed by granulocyte-colony stimulating factor to induce angiogenesis in patients with severe chronic ischaemic heart disease. Eur Heart J. 2006;27(15):1785–92. 16825290 10.1093/eurheartj/ehl117

[125] Ylä-Herttuala S, Baker AH. Cardiovascular Gene Therapy: Past, Present, and Future. Mol Ther. 2017;25(5):1095–106. 28389321 10.1016/j.ymthe.2017.03.027 PMC5417840

[126] Shi Y, Zhang H, Huang S, Yin L, Wang F, Luo P, Huang H. Epigenetic regulation in cardiovascular disease: mechanisms and advances in clinical trials. Signal Transduct Target Ther. 2022;7(1):200. 10.1038/s41392-022-01055-2. 35752619 10.1038/s41392-022-01055-2 PMC9233709

[127] Comerota AJ, Throm RC, Miller KA, Henry T, Chronos N, Laird J, et al. Naked plasmid DNA encoding fibroblast growth factor type 1 for the treatment of end-stage unreconstructible lower extremity ischemia: Preliminary results of a phase I trial. J Vasc Surg. 2002;35(5):930–6. 12021709 10.1067/mva.2002.123677

[128] Nikol S, Baumgartner I, Van Belle E, Diehm C, Visoná A, Capogrossi MC, et al. Therapeutic Angiogenesis With Intramuscular NV1FGF Improves Amputation-free Survival in Patients With Critical Limb Ischemia. Mol Ther. 2008;16(5):972–8. 28178491 10.1038/mt.2008.33

[129] Belch J, Hiatt WR, Baumgartner I, Driver IV, Nikol S, Norgren L, et al. Effect of fibroblast growth factor NV1FGF on amputation and death: a randomised placebo-controlled trial of gene therapy in critical limb ischaemia. The Lancet. 2011;377(9781):1929–37. 10.1016/S0140-6736(11)60394-2 21621834

[130] Morishita R, Makino H, Aoki M, Hashiya N, Yamasaki K, Azuma J, et al. Phase I/IIa Clinical Trial of Therapeutic Angiogenesis Using Hepatocyte Growth Factor Gene Transfer to Treat Critical Limb Ischemia. Arterioscler Thromb Vasc Biol. 2011;31(3):713–20. 21183732 10.1161/ATVBAHA.110.219550

[131] Powell RJ, Simons M, Mendelsohn FO, Daniel G, Henry TD, Koga M, et al. Results of a Double-Blind, Placebo-Controlled Study to Assess the Safety of Intramuscular Injection of Hepatocyte Growth Factor Plasmid to Improve Limb Perfusion in Patients With Critical Limb Ischemia. Circulation. 2008;118(1):58–65. 18559703 10.1161/CIRCULATIONAHA.107.727347

[132] Powell RJ, Goodney P, Mendelsohn FO, Moen EK, Annex BH. Safety and efficacy of patient specific intramuscular injection of HGF plasmid gene therapy on limb perfusion and wound healing in patients with ischemic lower extremity ulceration: Results of the HGF-0205 trial. J Vasc Surg. 2010;52(6):1525–30. 21146749 10.1016/j.jvs.2010.07.044 PMC5292269

[133] Rajagopalan S, Mohler ER, Lederman RJ, Mendelsohn FO, Saucedo JF, Goldman CK, et al. Regional Angiogenesis With Vascular Endothelial Growth Factor in Peripheral Arterial Disease. Circulation. 2003;108(16):1933–8. 14504183 10.1161/01.CIR.0000093398.16124.29

[134] Deev RV, Bozo IY, Mzhavanadze ND, Voronov DA, Gavrilenko AV, Chervyakov YV, et al. pCMV-vegf165 Intramuscular Gene Transfer is an Effective Method of Treatment for Patients With Chronic Lower Limb Ischemia. J Cardiovasc Pharmacol Ther. 2015;20(5):473–82. 25770117 10.1177/1074248415574336

[135] Shigematsu H, Yasuda K, Iwai T, Sasajima T, Ishimaru S, Ohashi Y, et al. Randomized, double-blind, placebo-controlled clinical trial of hepatocyte growth factor plasmid for critical limb ischemia. Gene Ther. 2010;17(9):1152–61. 20393508 10.1038/gt.2010.51

[136] Chervyakov YV, Staroverov IN, Vlasenko ON, Bozo IY, Isaev AA, Deev RV. [Five-year results of treating patients with chronic lower limb ischaemia by means of gene engineering]. Angiol Sosud Khir. 2016;22(4):38–44. 27935878

[137] Samuel TJ, Rosenberry RP, Lee S, Pan Z. Correcting Calcium Dysregulation in Chronic Heart Failure Using SERCA2a Gene Therapy. Int J Mol Sci. 2018;19(4):1086. 10.3390/ijms19041086. 29621141 10.3390/ijms19041086 PMC5979534

[138] Greenberg B, Butler J, Felker GM, Ponikowski P, Voors AA, Desai AS, et al. Calcium upregulation by percutaneous administration of gene therapy in patients with cardiac disease (CUPID 2): a randomised, multinational, double-blind, placebo-controlled, phase 2b trial. The Lancet. 2016;387(10024):1178–86. 10.1016/S0140-6736(16)00082-9 26803443

[139] Hammond HK, Penny WF, Traverse JH, Henry TD, Watkins MW, Yancy CW, et al. Intracoronary Gene Transfer of Adenylyl Cyclase 6 in Patients With Heart Failure: A Randomized Clinical Trial. JAMA Cardiol. 2016;1(2):163–71. 10.1001/jamacardio.2016.0008. 27437887 10.1001/jamacardio.2016.0008 PMC5535743

[140] Li M, Wang H, Tian L, Pang Z, Yang Q, Huang T, Fan J, Song L, Tong Y, Fan H. COVID-19 vaccine development: milestones, lessons and prospects. Signal Transduct Target Ther. 2022;7(1):146. 10.1038/s41392-022-00996-y. 35504917 10.1038/s41392-022-00996-y PMC9062866

[141] Brown MJ, Coltart J, Gunewardena K, Ritter JM, Auton TR, Glover JF. Randomized double-blind placebo-controlled study of an angiotensin immunotherapeutic vaccine (PMD3117) in hypertensive subjects. Clin Sci (London, England : 1979). 2004;107(2):167–73. 10.1042/CS20030381 15040783

[142] Koriyama H, Nakagami H, Nakagami F, Osako MK, Kyutoku M, Shimamura M, et al. Long-Term Reduction of High Blood Pressure by Angiotensin II DNA Vaccine in Spontaneously Hypertensive Rats. Hypertension. 2015;66(1):167–74. 26015450 10.1161/HYPERTENSIONAHA.114.04534

[143] Tokgözoğlu L, Libby P. The dawn of a new era of targeted lipid-lowering therapies. Eur Heart J. 2022;43(34):3198–208. 35051271 10.1093/eurheartj/ehab841 PMC9448630

[144] Egli M, Manoharan M. Chemistry, structure and function of approved oligonucleotide therapeutics. Nucleic Acids Res. 2023;51(6):2529–73. 36881759 10.1093/nar/gkad067 PMC10085713

[145] Ray KK, Kallend D, Leiter LA, Raal FJ, Koenig W, Jaros MJ, et al. Effect of inclisiran on lipids in primary prevention: the ORION-11 trial. Eur Heart J. 2022;43(48):5047–57. 36331315 10.1093/eurheartj/ehac615 PMC9769955

[146] Tsimikas S, Karwatowska-Prokopczuk E, Gouni-Berthold I, Tardif J-C, Baum SJ, Steinhagen-Thiessen E, et al. Lipoprotein(a) Reduction in Persons with Cardiovascular Disease. N Engl J Med. 2020;382(3):244–55. 31893580 10.1056/NEJMoa1905239

[147] Musunuru K, Chadwick AC, Mizoguchi T, Garcia SP, DeNizio JE, Reiss CW, et al. In vivo CRISPR base editing of PCSK9 durably lowers cholesterol in primates. Nature. 2021;593(7859):429–34. 34012082 10.1038/s41586-021-03534-y

[148] Scheltens P, De Strooper B, Kivipelto M, Holstege H, Chételat G, Teunissen CE, et al. Alzheimer’s disease. Lancet (London, England). 2021;397(10284):1577–90. 33667416 10.1016/S0140-6736(20)32205-4 PMC8354300

[149] Koutsodendris N, Nelson MR, Rao A, Huang Y. Apolipoprotein E and Alzheimer’s Disease: Findings, Hypotheses, and Potential Mechanisms. Annu Rev Pathol. 2022;17(1):73–99. 34460318 10.1146/annurev-pathmechdis-030421-112756

[150] Rosenberg JB, Kaplitt MG, De BP, Chen A, Flagiello T, Salami C, et al. AAVrh.10-Mediated APOE2 Central Nervous System Gene Therapy for APOE4-Associated Alzheimer's Disease. Human Gene Therapy Clin Dev. 2018;29(1):24–47. 10.1089/humc.2017.231 PMC5870071 29409358

[151] Rafii MS, Tuszynski MH, Thomas RG, Barba D, Brewer JB, Rissman RA, Siffert J, Aisen PS. AAV2-NGF Study Team. Adeno-Associated Viral Vector (Serotype 2)-Nerve Growth Factor for Patients With Alzheimer Disease: A Randomized Clinical Trial. JAMA Neurol. 2018;75(7):834-41. 10.1001/jamaneurol.2018.0233. 10.1001/jamaneurol.2018.0233 PMC5885277 29582053

[152] De Plano LM, Calabrese G, Conoci S, Guglielmino SPP, Oddo S, Caccamo A. Applications of CRISPR-Cas9 in Alzheimer’s Disease and Related Disorders. Int J Mol Sci. 2022;23(15):8714. 10.3390/ijms23158714. 35955847 10.3390/ijms23158714 PMC9368966

[153] Ye H, Robak LA, Yu M, Cykowski M, Shulman JM. Genetics and Pathogenesis of Parkinson’s Syndrome. Annu Rev Pathol. 2023;18(1):95–121. 36100231 10.1146/annurev-pathmechdis-031521-034145 PMC10290758

[154] Bartus RT, Baumann TL, Siffert J, Herzog CD, Alterman R, Boulis N, et al. Safety/feasibility of targeting the substantia nigra with AAV2-neurturin in Parkinson patients. Neurology. 2013;80(18):1698–701. 10.1212/WNL.0b013e3182904faa. Epub 2013 Apr 10. 23576625 10.1212/WNL.0b013e3182904faa PMC3716474

[155] Christine CW, Bankiewicz KS, Van Laar AD, Richardson RM, Ravina B, Kells AP, et al. Magnetic resonance imaging–guided phase 1 trial of putaminal AADC gene therapy for Parkinson’s disease. Ann Neurol. 2019;85(5):704–14. 30802998 10.1002/ana.25450 PMC6593762

[156] Palfi S, Gurruchaga JM, Ralph GS, Lepetit H, Lavisse S, Buttery PC, et al. Long-term safety and tolerability of ProSavin, a lentiviral vector-based gene therapy for Parkinson’s disease: a dose escalation, open-label, phase 1/2 trial. The Lancet. 2014;383(9923):1138–46. 10.1016/S0140-6736(13)61939-X 24412048

[157] LeWitt PA, Rezai AR, Leehey MA, Ojemann SG, Flaherty AW, Eskandar EN, et al. AAV2-GAD gene therapy for advanced Parkinson’s disease: a double-blind, sham-surgery controlled, randomised trial. The Lancet Neurology. 2011;10(4):309–19. 21419704 10.1016/S1474-4422(11)70039-4

[158] Blauwendraat C, Reed X, Krohn L, Heilbron K, Bandres-Ciga S, Tan M, et al. Genetic modifiers of risk and age at onset in GBA associated Parkinson’s disease and Lewy body dementia. Brain : a journal of neurology. 2020;143(1):234–48. 31755958 10.1093/brain/awz350 PMC6935749

[159] Lu NN, Tan C, Sun NH, Shao LX, Liu XX, Gao YP, et al. Cholinergic Grb2-Associated-Binding Protein 1 Regulates Cognitive Function. Cerebral Cortex (New York, NY : 1991). 2018;28(7):2391–404. 10.1093/cercor/bhx141 PMC7189983 28591834

[160] Cao R, Chen C, Wen J, Zhao W, Zhang C, Sun L, Yuan L, Wu C, Shan L, Xi M, Sun H. Recent advances in targeting leucine-rich repeat kinase 2 as a potential strategy for the treatment of Parkinson's disease. Bioorg Chem. 2023;141:106906. 10.1016/j.bioorg.2023.106906. Epub 2023 Oct 7. 10.1016/j.bioorg.2023.106906 37837728

[161] Feldman EL, Goutman SA, Petri S, Mazzini L, Savelieff MG, Shaw PJ, et al. Amyotrophic lateral sclerosis. The Lancet. 2022;400(10360):1363–80. 10.1016/S0140-6736(22)01272-7 PMC10089700 36116464

[162] Abati E, Bresolin N, Comi G, Corti S. Silence superoxide dismutase 1 (SOD1): a promising therapeutic target for amyotrophic lateral sclerosis (ALS). Expert Opin Ther Targets. 2020;24(4):295–310. 32125907 10.1080/14728222.2020.1738390

[163] Miller TM, Pestronk A, David W, Rothstein J, Simpson E, Appel SH, et al. An antisense oligonucleotide against SOD1 delivered intrathecally for patients with SOD1 familial amyotrophic lateral sclerosis: a phase 1, randomised, first-in-man study. The Lancet Neurology. 2013;12(5):435–42. 23541756 10.1016/S1474-4422(13)70061-9 PMC3712285

[164] Tran H, Moazami MP, Yang H, McKenna-Yasek D, Douthwright CL, Pinto C, et al. Suppression of mutant C9orf72 expression by a potent mixed backbone antisense oligonucleotide. Nat Med. 2022;28(1):117–24. 34949835 10.1038/s41591-021-01557-6 PMC8861976

[165] Glass JD, Dewan R, Ding J, Gibbs JR, Dalgard C, Keagle PJ, et al. ATXN2 intermediate expansions in amyotrophic lateral sclerosis. Brain : a journal of neurology. 2022;145(8):2671–6. 35521889 10.1093/brain/awac167 PMC9890463

[166] Suk TR, Rousseaux MWC. The role of TDP-43 mislocalization in amyotrophic lateral sclerosis. Mol Neurodegener. 2020;15(1):45. 32799899 10.1186/s13024-020-00397-1 PMC7429473

[167] Watanabe R, Higashi S, Nonaka T, Kawakami I, Oshima K, Niizato K, et al. Intracellular dynamics of Ataxin-2 in the human brains with normal and frontotemporal lobar degeneration with TDP-43 inclusions. Acta Neuropathol Commun. 2020;8(1):176. 33115537 10.1186/s40478-020-01055-9 PMC7594343

[168] Wan PK-T, Ryan AJ, Seymour LW. Beyond cancer cells: Targeting the tumor microenvironment with gene therapy and armed oncolytic virus. Mole Therapy. 2021;29(5):1668–82. 10.1016/j.ymthe.2021.04.015 PMC8116634 33845199

[169] Mai D, June CH, Sheppard NC. In vivo gene immunotherapy for cancer. Sci Transl Med. 2022;14(670):eabo3603. 36350990 10.1126/scitranslmed.abo3603

[170] Singh V, Khan N, Jayandharan GR. Vector engineering, strategies and targets in cancer gene therapy. Cancer Gene Ther. 2021;29(5):402–17. 33859378 10.1038/s41417-021-00331-7

[171] Roetman JJ, Apostolova MKI, Philip M. Viral and cellular oncogenes promote immune evasion. Oncogene. 2022;41(7):921–9. 35022539 10.1038/s41388-021-02145-1 PMC8851748

[172] Duarte S, Carle G, Faneca H, de Lima MC, Pierrefite-Carle V. Suicide gene therapy in cancer: where do we stand now? Cancer Lett. 2012;324(2):160–70. 22634584 10.1016/j.canlet.2012.05.023

[173] Fillat C, Carrió M, Cascante A, Sangro B. Suicide gene therapy mediated by the Herpes Simplex virus thymidine kinase gene/Ganciclovir system: fifteen years of application. Curr Gene Ther. 2003;3(1):13–26. 12553532 10.2174/1566523033347426

[174] Macleod K. Tumor suppressor genes. Curr Opin Genet Dev. 2000;10(1):81–93. 10679386 10.1016/S0959-437X(99)00041-6

[175] Wilson JM. Gendicine: the first commercial gene therapy product. Hum Gene Ther. 2005;16(9):1014–5. 16149899 10.1089/hum.2005.16.1014

[176] Kelly E, Russell SJ. History of oncolytic viruses: genesis to genetic engineering. Molecular therapy : the journal of the American Society of Gene Therapy. 2007;15(4):651–9. 17299401 10.1038/sj.mt.6300108

[177] Andtbacka RH, Kaufman HL, Collichio F, Amatruda T, Senzer N, Chesney J, et al. Talimogene Laherparepvec Improves Durable Response Rate in Patients With Advanced Melanoma. Journal of clinical oncology : official journal of the American Society of Clinical Oncology. 2015;33(25):2780–8. 26014293 10.1200/JCO.2014.58.3377

[178] Oh E, Choi IK, Hong J, Yun CO. Oncolytic adenovirus coexpressing interleukin-12 and decorin overcomes Treg-mediated immunosuppression inducing potent antitumor effects in a weakly immunogenic tumor model. Oncotarget. 2017;8(3):4730–46. 28002796 10.18632/oncotarget.13972 PMC5354867

[179] Wenthe J, Naseri S, Hellström AC, Wiklund HJ, Eriksson E, Loskog A. Immunostimulatory oncolytic virotherapy for multiple myeloma targeting 4–1BB and/or CD40. Cancer Gene Ther. 2020;27(12):948–59. 32355275 10.1038/s41417-020-0176-9 PMC7725669

[180] Ding Y, Fan J, Deng L, Huang B, Zhou B. Antitumor efficacy of cytosine deaminase-armed vaccinia virus plus 5-fluorocytosine in colorectal cancers. Cancer Cell Int. 2020;20:243. 32549790 10.1186/s12935-020-01340-6 PMC7296660

[181] Havunen R, Siurala M, Sorsa S, Grönberg-Vähä-Koskela S, Behr M, Tähtinen S, et al. Oncolytic Adenoviruses Armed with Tumor Necrosis Factor Alpha and Interleukin-2 Enable Successful Adoptive Cell Therapy. Molecular therapy oncolytics. 2017;4:77–86. 28345026 10.1016/j.omto.2016.12.004 PMC5363700

[182] Liang M. Oncorine, the World First Oncolytic Virus Medicine and its Update in China. Curr Cancer Drug Targets. 2018;18(2):171–6. 29189159 10.2174/1568009618666171129221503

[183] Greig SL. Talimogene Laherparepvec: First Global Approval. Drugs. 2016;76(1):147–54. 26620366 10.1007/s40265-015-0522-7

[184] Lang F, Schrörs B, Löwer M, Türeci Ö, Sahin U. Identification of neoantigens for individualized therapeutic cancer vaccines. Nat Rev Drug Discovery. 2022;21(4):261–82. 35105974 10.1038/s41573-021-00387-y PMC7612664

[185] Sahin U, Oehm P, Derhovanessian E, Jabulowsky RA, Vormehr M, Gold M, et al. An RNA vaccine drives immunity in checkpoint-inhibitor-treated melanoma. Nature. 2020;585(7823):107–12. 32728218 10.1038/s41586-020-2537-9

[186] Maude SL, Laetsch TW, Buechner J, Rives S, Boyer M, Bittencourt H, et al. Tisagenlecleucel in Children and Young Adults with B-Cell Lymphoblastic Leukemia. N Engl J Med. 2018;378(5):439–48. 29385370 10.1056/NEJMoa1709866 PMC5996391

[187] Labanieh L, Mackall CL. CAR immune cells: design principles, resistance and the next generation. Nature. 2023;614(7949):635–48. 36813894 10.1038/s41586-023-05707-3

[188] Pan K, Farrukh H, Chittepu VCSR, Xu H, Pan CX, Zhu Z. CAR race to cancer immunotherapy: from CAR T, CAR NK to CAR macrophage therapy. J Exp Clin Cancer Res. 2022;41(1):119. 10.1186/s13046-022-02327-z. 35361234 10.1186/s13046-022-02327-z PMC8969382

[189] Cappell KM, Kochenderfer JN. Long-term outcomes following CAR T cell therapy: what we know so far. Nat Rev Clin Oncol. 2023;20(6):359–71. 37055515 10.1038/s41571-023-00754-1 PMC10100620

[190] Maalej KM, Merhi M, Inchakalody VP, Mestiri S, Alam M, Maccalli C, Cherif H, Uddin S, Steinhoff M, Marincola FM, Dermime S. CAR-cell therapy in the era of solid tumor treatment: current challenges and emerging therapeutic advances. Mol Cancer. 2023;22(1):20. 10.1186/s12943-023-01723-z. 36717905 10.1186/s12943-023-01723-z PMC9885707

[191] Maalej KM, Merhi M, Inchakalody VP, Mestiri S, Alam M, Maccalli C, et al. CAR-cell therapy in the era of solid tumor treatment: current challenges and emerging therapeutic advances. Mol Cancer. 2023;22(1):20. 36717905 10.1186/s12943-023-01723-z PMC9885707

[192] Katti A, Diaz BJ, Caragine CM, Sanjana NE, Dow LE. CRISPR in cancer biology and therapy. Nat Rev Cancer. 2022;22(5):259–79. 35194172 10.1038/s41568-022-00441-w

[193] Eizirik DL, Pasquali L, Cnop M. Pancreatic β-cells in type 1 and type 2 diabetes mellitus: different pathways to failure. Nat Rev Endocrinol. 2020;16(7):349–62. 32398822 10.1038/s41574-020-0355-7

[194] Redondo MJ, Gignoux CR, Dabelea D, Hagopian WA, Onengut-Gumuscu S, Oram RA, et al. Type 1 diabetes in diverse ancestries and the use of genetic risk scores. Lancet Diabetes Endocrinol. 2022;10(8):597–608. 35724677 10.1016/S2213-8587(22)00159-0 PMC10024251

[195] Morris AP, Voight BF, Teslovich TM, Ferreira T, Segrè AV, Steinthorsdottir V, et al. Large-scale association analysis provides insights into the genetic architecture and pathophysiology of type 2 diabetes. Nat Genet. 2012;44(9):981–90. 22885922 10.1038/ng.2383 PMC3442244

[196] Chen J, Sun M, Adeyemo A, Pirie F, Carstensen T, Pomilla C, et al. Genome-wide association study of type 2 diabetes in Africa. Diabetologia. 2019;62(7):1204–11. 31049640 10.1007/s00125-019-4880-7 PMC6560001

[197] Kooner JS, Saleheen D, Sim X, Sehmi J, Zhang W, Frossard P, et al. Genome-wide association study in individuals of South Asian ancestry identifies six new type 2 diabetes susceptibility loci. Nat Genet. 2011;43(10):984–9. 21874001 10.1038/ng.921 PMC3773920

[198] Sladek R, Rocheleau G, Rung J, Dina C, Shen L, Serre D, et al. A genome-wide association study identifies novel risk loci for type 2 diabetes. Nature. 2007;445(7130):881–5. 17293876 10.1038/nature05616

[199] Matsuoka TA, Kawashima S, Miyatsuka T, Sasaki S, Shimo N, Katakami N, et al. Mafa Enables Pdx1 to Effectively Convert Pancreatic Islet Progenitors and Committed Islet α-Cells Into β-Cells In Vivo. Diabetes. 2017;66(5):1293–300. 28223284 10.2337/db16-0887 PMC5399608

[200] Xiao X, Guo P, Shiota C, Zhang T, Coudriet GM, Fischbach S, et al. Endogenous Reprogramming of Alpha Cells into Beta Cells, Induced by Viral Gene Therapy. Reverses Autoimmune Diabetes Cell Stem Cell. 2018;22(1):78-90.e4. 29304344 10.1016/j.stem.2017.11.020 PMC5757249

[201] Osipovich AB, Magnuson MA. Alpha to Beta Cell Reprogramming: Stepping toward a New Treatment for Diabetes. Cell Stem Cell. 2018;22(1):12–3. 29304337 10.1016/j.stem.2017.12.012

[202] Genprex. Clinical trials R&D Pipeline [Available from: https://www.genprex.com/clinical-trials/pipeline/.

[203] Therapeutics C. CRISPR Therapeutics and ViaCyte, Inc. Announce First Patient Dosed in Phase 1 Clinical Trial of Novel Gene-Edited Cell Replacement Therapy for Treatment of Type 1 Diabetes (T1D) [Available from: https://crisprtx.com/about-us/press-releases-and-presentations/crispr-therapeutics-and-viacyte-inc-announce-first-patient-dosed-in-phase-1-clinical-trial-of-novel-gene-edited-cell-replacement-therapy-for-treatment-of-type-1-diabetes-t1d.

[204] Zammit NW, Seeberger KL, Zamerli J, Walters SN, Lisowski L, Korbutt GS, Grey ST. Selection of a novel AAV2/TNFAIP3 vector for local suppression of islet xenograft inflammation. Xenotransplantation. 2021;28(3):e12669. 10.1111/xen.12669. Epub 2020 Dec 14. 10.1111/xen.12669 33316848

[205] Zammit NW, Walters SN, Seeberger KL, O'Connell PJ, Korbutt GS, Grey ST. A20 as an immune tolerance factor can determine islet transplant outcomes. JCI Insight. 2019;4(21):e131028. 10.1172/jci.insight.131028. 10.1172/jci.insight.131028 PMC6948770 31581152

[206] Hinney A, Körner A, Fischer-Posovszky P. The promise of new anti-obesity therapies arising from knowledge of genetic obesity traits. Nat Rev Endocrinol. 2022;18(10):623–37. 35902734 10.1038/s41574-022-00716-0 PMC9330928

[207] Wang CH, Lundh M, Fu A, Kriszt R, Huang TL, Lynes MD, et al. CRISPRengineered human brown-like adipocytes prevent diet-induced obesity and ameliorate metabolic syndrome in mice. Sci Transl Med. 2020;12(558):eaaz8664. 10.1126/scitranslmed.aaz8664. 10.1126/scitranslmed.aaz8664 PMC7704293 32848096

[208] Tang R, Harasymowicz NS, Wu CL, Collins KH, Choi YR, Oswald SJ, et al. Gene therapy for follistatin mitigates systemic metabolic inflammation and post-traumatic arthritis in high-fat diet-induced obesity. Sci Adv. 2020;6(19):eaaz7492. 32426485 10.1126/sciadv.aaz7492 PMC7209997

[209] Akbari P, Gilani A, Sosina O, Kosmicki JA, Khrimian L, Fang YY, et al. Sequencing of 640,000 exomes identifies GPR75 variants associated with protection from obesity. Science. 2021;373(6550):eabf8683. 10.1126/science.abf8683. 10.1126/science.abf8683 PMC10275396 34210852

[210] Scherer HU, Häupl T, Burmester GR. The etiology of rheumatoid arthritis. J Autoimmun. 2020;110:102400. 10.1016/j.jaut.2019.102400. Epub 2020 Jan 22. 10.1016/j.jaut.2019.102400 31980337

[211] Ding Q, Hu W, Wang R, Yang Q, Zhu M, Li M, Cai J, Rose P, Mao J, Zhu YZ. Signaling pathways in rheumatoid arthritis: implications for targeted therapy. Signal Transduct Target Ther. 2023;8(1):68. 10.1038/s41392-023-01331-9. 36797236 10.1038/s41392-023-01331-9 PMC9935929

[212] Han H, Xing J, Chen W, Jia J, Li Q. Fluorinated polyamidoamine dendrimer-mediated miR-23b delivery for the treatment of experimental rheumatoid arthritis in rats. Nat Commun. 2023;14(1):944. 10.1038/s41467-023-36625-7. 36805456 10.1038/s41467-023-36625-7 PMC9941585

[213] Wang F, Yu J, Wang Y, Jiang Y, Guo N, Zhang W. Combination therapy with TNFR-Fc and CTLA4-FasL using the recombinant adeno-associated virus potently suppresses adjuvant-induced arthritis in rats. Appl Microbiol Biotechnol. 2015;99(15):6327–37. 25707864 10.1007/s00253-015-6459-7

[214] Shaw AM, Qasem A, Naser SA. Modulation of PTPN2/22 Function by Spermidine in CRISPR-Cas9-Edited TCells Associated with Crohn’s Disease and Rheumatoid Arthritis. Int J Mol Sci. 2021;22(16):8883. 10.3390/ijms22168883. 34445589 10.3390/ijms22168883 PMC8396355

[215] Lee MH, Shin JI, Yang JW, Lee KH, Cha DH, Hong JB, Park Y, Choi E, Tizaoui K, Koyanagi A, Jacob L, Park S, Kim JH, Smith L. Genome Editing Using CRISPR-Cas9 and Autoimmune Diseases: A Comprehensive Review. Int J Mol Sci. 2022;23(3):1337. 10.3390/ijms23031337. 35163260 10.3390/ijms23031337 PMC8835887

[216] Duan WL, Zhang LN, Bohara R, Martin-Saldaña S, Yang F, Zhao YY, et al. Adhesive hydrogels in osteoarthritis: from design to application. Mil Med Res. 2023;10(1):4. 36710340 10.1186/s40779-022-00439-3 PMC9885614

[217] Cho Y, Jeong S, Kim H, Kang D, Lee J, Kang SB, et al. Disease-modifying therapeutic strategies in osteoarthritis: current status and future directions. Exp Mol Med. 2021;53(11):1689–96. 34848838 10.1038/s12276-021-00710-y PMC8640059

[218] Mattiotti A, Prakash S, Barnett P, van den Hoff MJB. Follistatin-like 1 in development and human diseases. Cell Mol Life Sci. 2018;75(13):2339–54. 29594389 10.1007/s00018-018-2805-0 PMC5986856

[219] Zhao L, Huang J, Fan Y, Li J, You T, He S, et al. Exploration of CRISPR/Cas9-based gene editing as therapy for osteoarthritis. Ann Rheum Dis. 2019;78(5):676–82. 30842121 10.1136/annrheumdis-2018-214724 PMC6621547

[220] Kiriakidou M, Ching CL. Systemic Lupus Erythematosus. Ann Intern Med. 2020;172(11):Itc81-itc96. 32479157 10.7326/AITC202006020

[221] Werling DM, Brand H, An JY, Stone MR, Zhu L, Glessner JT, et al. An analytical framework for whole-genome sequence association studies and its implications for autism spectrum disorder. Nat Genet. 2018;50(5):727–36. 29700473 10.1038/s41588-018-0107-y PMC5961723

[222] Julian TH, Boddy S, Islam M, Kurz J, Whittaker KJ, Moll T, et al. A review of Mendelian randomization in amyotrophic lateral sclerosis. Brain : a journal of neurology. 2022;145(3):832–42. 34791088 10.1093/brain/awab420 PMC9050546

[223] Silva GFS, Fagundes TP, Teixeira BC, Chiavegatto Filho ADP. Machine Learning for Hypertension Prediction: a Systematic Review. Curr Hypertens Rep. 2022;24(11):523–33. 35731335 10.1007/s11906-022-01212-6

[224] Zhang L, Li Z, Skrzypczynska KM, Fang Q, Zhang W, O’Brien SA, et al. Single-Cell Analyses Inform Mechanisms of Myeloid-Targeted Therapies in Colon Cancer. Cell. 2020;181(2):442-59.e29. 32302573 10.1016/j.cell.2020.03.048

[225] Santiago-Ortiz J, Ojala DS, Westesson O, Weinstein JR, Wong SY, Steinsapir A, et al. AAV ancestral reconstruction library enables selection of broadly infectious viral variants. Gene Ther. 2015;22(12):934–46. 26186661 10.1038/gt.2015.74 PMC4509550

[226] Bentler M, Hardet R, Ertelt M, Rudolf D, Kaniowska D, Schneider A, et al. Modifying immune responses to adeno-associated virus vectors by capsid engineering. Molecular therapy Methods & clinical development. 2023;30:576–92. 37693943 10.1016/j.omtm.2023.08.015 PMC10485635

